# The NFIL3-MYC signaling axis: a novel therapeutic target and prognostic marker in high-risk multiple myeloma

**DOI:** 10.1186/s40364-026-01018-7

**Published:** 2026-10-09

**Authors:** Daniela Simone Maichl, Wen-Hui Cheng, Poonam Kumari, Julius Arthur Kirner, Stefanie Schmidt, Carsten Patrick Ade, Mengshi Yang, Malik Salman Haider, Yuquan Chi, Robert Luxenhofer, Lyudmyla Taranets, Melanie Haffner-Luntzer, Verena Fischer, Anita Ignatius, Ellen Leich, Gerardo Zapata, Nikita Popov, Franziska Jundt

**Affiliations:** 1 Department of Internal Medicine II, University Hospital Würzburg, Oberdürrbacher Straße 6, 97080 Würzburg, Germany; 2 Department of General, Visceral, Transplant, Vascular and Pediatric Surgery, University Hospital Würzburg, Oberdürrbacher Straße 6, 97080 Würzburg, Germany; 3 Department of Biochemistry and Molecular Biology, Biocenter, University of Würzburg, Am Hubland, 97074 Würzburg, Germany; 4 Functional Polymer Materials, Chair for Advanced Materials Synthesis, Institute for Functional Materials and Biofabrication, Department of Chemistry and Pharmacy, University of Würzburg, Röntgenring 11, 97070 Würzburg, Germany; 5 Department of Ophthalmology, University Hospital Würzburg, Josef- Schneider-Straße 11, 97080 Würzburg, Germany; 6 Soft Matter Chemistry, Department of Chemistry and Helsinki Institute of Sustainability Science, Faculty of Science, University of Helsinki, PB 55, Helsinki, 00014 Finland; 7 Department of Internal Medicine VIII, University Hospital Tübingen, Otfried-Müller-Strasse 14, 72076 Tübingen, Germany; 8 Institute of Orthopedic Research and Biomechanics, University Medical Center Ulm, Helmholtzstraße 14, 89081 Ulm, Germany; 9 Institute of Pathology, University of Würzburg, Würzburg, Germany; 10 Canadian Centre for Computational Genomics, McGill University, Montréal, Québec H3A22 2R7 Canada; 11 Victor Phillip Dahdaleh Institute of Genomic Medicine at McGill University, 24 Québec, Montréal, H3A 0G1 Canada

**Keywords:** Multi-refractory multiple myeloma, NFIL3, c-MYC, Raddeanin A, High-risk cytogenetics

## Abstract

**Background:**

Drug resistance causes tumor relapse, limits the outcomes of patients with high-risk multiple myeloma (MM), and requires the development of targeted therapeutics. Increased Notch signaling promotes drug resistance in high-risk del(17p)/TP53-deficient clonogenic MM cells, highlighting the pathway and its downstream effectors as rational therapeutic targets in aggressive MM. However, the identification of selective, safe, and efficacious inhibitors targeting the Notch pathway remains a substantial challenge.

**Methods:**

To identify novel druggable Notch effectors, we performed transcriptome profiling of Notch-depleted MM cells and selected potential candidates. We applied functional high-throughput shRNA library screens targeting these genes in MM cells with high Notch activity and under drug selection. Through bioinformatic analysis, we selected NFIL3 and validated its mechanistic specificity using ChIP-seq analysis, immunoblotting, cell viability and growth assays. We targeted NFIL3 via the oleanane-type triterpenoid saponin Raddeanin A (RA) and tested its efficacy in vivo using the murine MOPC.315.BM.Luc.eGFP-*mCrbn*^*I391V*^ model. To improve efficacy of RA in vivo, we developed drug-loaded nanocarriers based on polymer micelles. Using patient MM cells with high-risk cytogenetic abnormalities and post B-cell maturation antigen (BCMA)-directed immunotherapy, we demonstrated efficacy of RA to circumvent multi-drug resistance. To determine the prognostic significance of NFIL3, we correlated its transcriptional expression with survival data and cytogenetic abnormalities of two patient cohorts.

**Results:**

In functional genomics screens, we identified NFIL3 as a critical therapeutic vulnerability in high-risk, multidrug-resistant MM. Mechanistic studies silencing NFIL3 revealed that NFIL3 acts as a repressor of the MYC oncogenic driver in MM cells. RA collapsed the MYC-driven transcriptional program essential for MM cell survival and abrogated multidrug resistance of human and murine MM cells in vitro and in vivo. Importantly, the drug RA demonstrated potent anti-myeloma activity in patient-derived cells with high-risk cytogenetics following failure of BCMA-directed immunotherapies. Furthermore, high *NFIL3* expression was associated with high-risk cytogenetic abnormalities and extramedullary disease and was verified as an independent prognostic marker for overall survival in MM patients.

**Conclusions:**

RA targets the NFIL3-MYC axis and demonstrates potent anti-myeloma activity in vivo and in patient-derived high-risk MM cells following failure of BCMA-directed immunotherapies, providing a potent salvage strategy for multi-refractory disease.

**Supplementary Information:**

The online version contains supplementary material available at https://doi.org/10.1186/s40364-026-01018-7.

## Background

Multiple myeloma (MM) is a biologically heterogeneous clonal plasma cell malignancy with highly variable clinical outcomes [1]. Despite major therapeutic advances, a subset of patients with high-risk cytogenetic disease shows early relapse, an aggressive disease course, and still has a markedly inferior survival [1]. High-risk cytogenetic disease is defined by adverse genomic abnormalities including the primary t(4;14), t(14;16), t(14;20), and the secondary abnormalities del(17p), *TP53* mutations, and gain or amplification of 1q (gain/amp(1q)) [2]. In this subgroup, drug resistance is a central driver of treatment failure and is further aggravated in patients with extramedullary disease (EMD) or after failure of current immunotherapies such as post B-cell maturation antigen (BCMA)-directed immunotherapy [2–4]. Therefore, identifying molecular drivers beyond standard cytogenetic classification is essential for the development of new targeted salvage strategies in the high-risk subgroup.

Notch signaling is an important driver of the pathobiology of aggressive MM through tumor-intrinsic and microenvironment-dependent mechanisms [5–13]. Early studies showed that Jagged-dependent Notch activation promotes MM cell proliferation and that stromal Notch1 signaling mediates de novo resistance to anti-myeloma therapy, whereas pharmacologic inhibition of the pathway induces apoptosis and restores chemosensitivity [10, 11, 14]. Subsequent transcriptomic and functional analyses extended these observations to clinically aggressive disease states, demonstrating enrichment of Notch-pathway components in high-risk molecular subgroups. These groups included musculoaponeurotic fibrosarcoma oncogene family (MAF)-translocated MM in t(14;16) and t(14;20) as well as primary plasma cell leukemia, linking Notch activation to malignant progression and adverse-risk biology [15]. Notably, a direct functional connection to high-risk cytogenetic MM has been demonstrated in del(17p)/TP53-deficient disease, in which p53 loss activates Notch signaling and renders clonogenic MM cells selectively vulnerable to the depletion of the Notch target HES1 or to the pharmacologic inhibition of the pathway by γ-secretase inhibitors [5]. In parallel, aberrant Notch signaling has been implicated in acquired treatment resistance, including Jagged1-driven bortezomib resistance and a NOTCH3-CXCL12 signaling axis that promotes tumor microenvironment-mediated chemoresistance [9, 16]. Collectively, these findings show that aberrant Notch signaling in MM is functionally involved in high-risk, drug-resistant disease, underscoring the therapeutic relevance of targeting this pathway and its downstream effectors in aggressive MM.

Direct clinical blockade of Notch has been difficult because broad pathway inhibition causes substantial on-target toxicity, particularly gastrointestinal toxicity, as seen with the γ-secretase inhibitor MK-0752 and the anti-Notch1 antibody brontictuzumab, in which diarrhea was a prominent dose-limiting or treatment-related adverse event [17, 18]. In MM, γ-secretase inhibitors are currently being explored at low doses to increase membrane BCMA and support BCMA-directed therapy, as shown preclinically and in early clinical testing of crenigacestat plus chimeric antigen receptor T cell (CAR-T) immunotherapy [19, 20]. However, these low doses of γ-secretase inhibitors are not sufficient to kill MM cells. To efficiently target the Notch pathway with refined specificity and less toxicity in MM cells, the identification of novel druggable Notch effectors is essential. Using functional genomics screens with shRNA libraries against druggable Notch effectors, we identified the NFIL3-MYC axis as a novel vulnerability in high-risk, multi-refractory MM that can be efficiently targeted by Raddeanin A (RA) [21] as a potent novel salvage strategy.

## Methods

### Cell lines

Human MM cell lines and HEK293T cells were cultured using standard conditions in 5% CO_2_ at 37 °C in RPMI 1640 or Dulbecco’s modified Eagle’s medium (Thermo Fisher Scientific, Darmstadt, Germany), supplemented with 10% FCS (Anprotec, Bruckberg, Germany), 1% penicillin/streptomycin, and 1% sodium pyruvate (Thermo Fisher Scientific). MOPC.315.BM.Luc.eGFP-*mCrbn*^*I391V*^ cells were cultured in RPMI 1640 medium with 10% FCS, 1% MEM non-essential amino acid (Sigma-Aldrich, Steinheim, Germany), 50 µM I-thioglycerol (Sigma-Aldrich), and 9.6 µg/ml Gentamicin (Biowest, Nuaillé, France).

### Patient samples

Mononuclear cells were isolated from bone marrow aspirates by diluting the samples in Red Blood Cell lysis solution (Miltenyi Biotec, Bergisch Gladbach, Germany), followed by density gradient centrifugation using Histopaque^®^-1077 Hybri-Max™ solution (Sigma-Aldrich). Lastly, CD138^+^ and CD138^−^ cells were separated using CD138 MicroBeads and large cell separation columns (Miltenyi Biotec). Subsequently, CD138^+^ cells were resuspended in RPMI 1640 medium with 10% FCS, seeded at a density of 2 × 10^5^ cells/ml in a 96-well plate (100 µl/well), and immediately treated in triplicates. Purity of CD138^+^ cells was assessed via flow cytometry analysis (> 85%). This study was approved by the local Ethics Committee of the University of Würzburg (224/20-am). All patients signed written informed consent forms in accordance with the Declaration of Helsinki.

### Drugs

Stock solutions of Raddeanin A (TargetMol, Boston, MA, USA), lenalidomide (Len), melphalan (Mel), and bortezomib (Btz, Cayman Chemical, Ann Arbor, MI, USA) were prepared in DMSO at concentrations of 25 mM, 100 mM, 30 mM, 100 mM, aliquoted and stored at -80 °C. The combination index (CI) for two-drug combinations was calculated using CompuSyn according to the Chou-Talalay method. CI was calculated as CI = (D)_1_/(D_x_)_1_+(D)_2_/(D_x_)_2_, where (D)_1_ and (D)_2_ are the concentrations of drugs 1 and 2 in combination producing a given fractional effect (Fa), and (Dx)_1_ and (Dx)_2_ are the concentrations of the respective drugs administered individually that produce the same effect in reducing cell viability.

### Transcriptome profiling

RNA sequencing (RNAseq) analyses of MM.1S cells were performed after GSI XII (5 µM) or DMSO treatment for 24 h or *NOTCH1* knockdown using two different specific shRNAs (shN1 #1, target sequence: GAACGAGCATAGTCCAAAAA; shN1 #2, target sequence: GTAGTTGTTCGTTGGTTATA) cloned into the pLKO.1-puro vector (Addgene, Watertown, MA, USA) at the AgeI and EcoRI restriction sites, versus empty vector (EV).

Total RNA was extracted with the RNeasy Plus Mini kit (Qiagen, Hilden, Germany) and mRNA was isolated using the NEBNext Poly(A) mRNA Magnetic Isolation Module (New England Biolabs, Frankfurt, Germany). Libraries were prepared using the NEBNext Ultra II RNA Library Prep Kit for Illumina (New England Biolabs) and AMPure XP beads (Beckman Coulter, Beverly, MA, USA) for size selection. Samples were amplified and sample-specific indices (NEBNext Multiplex Oligos for Illumina, Dual Index Primer Set 1, Illumina, New England Biolabs) were introduced. After quantification and determination of library size using a 2100 Bioanalyzer System (Agilent Technologies, Santa Clara, CA, USA), the samples were sequenced on a NextSeq 500 System (Illumina, San Diego, CA, USA).

RNAseq data of our previously published validation cohort with 73 CD138-sorted samples from 52 patients (for patient characteristics see Table S3) was performed as previously described [22]. All methods were performed in accordance with the relevant guidelines and regulations. Informed consent was obtained from all subjects. From the Multiple Myeloma Research Foundation one RNAseq dataset (MMRF CoMMpass study (IA15); 921 samples from 806 patients) was available. We used the R package “MMRFBiolinks” [23] to access and download the MMRF CoMMpass dataset. Gene expression data, as transcripts per million (TPM), were derived from Salmon pseudo-quantifications [24]. Patients were included if they had paired longitudinal samples corresponding to both baseline and confirmed disease progression. Only patients with at least two valid BM samples (baseline and progression) were retained.

### Lentivirus production and transduction

Lentiviruses were produced and used for transduction as previously described [8]. Briefly, HEK293T cells were co-transfected with a mix of 10 µg transfer plasmids, 5 µg packaging plasmid psPAX.2, and 2.5 µg envelope plasmid pMD2.G using the TransIT-Lenti transfection reagent (Mirus Bio, Madison, WI, USA). The virus-containing supernatant was harvested after 48 h, passed through a 0.45 μm filter, and immediately used for transduction. Cells were transduced by spinoculation (800 g, 32 °C, 1.5 h) in 15 ml falcon tubes containing 1 ml cell suspension (10^6^ cells), 1 ml virus supernatant, and polybrene (8 µg/ml final concentration; Sigma-Aldrich). Selection of stably transduced cells started after 24 h by adding the appropriate antibiotics to the cell culture medium (supplemental Table 1).

### Gene set enrichment analyses (GSEA)

Gene set enrichment analyses (GSEA) were performed with signal2noise metric, 1,000 permutations [25]. Gene sets of *NOTCH1* inhibited genes (GSI versus DMSO: *Q* < 0.05 and log_2_FC < 0) and *NOTCH1* knockdown genes (shN1 #1 versus EV, shN1 #2 versus EV, r: Q < 0.05 and log_2_FC < 0) were defined and used as a gene set database for GSEA.

### Functional genomics screens

Transcriptome data was filtered for Notch-regulated genes. Following evaluation by their strength and consistency of regulation, 40 Notch target genes were chosen for the focused shRNA screens (supplemental Table 2). A custom shRNA library comprising five shRNA sequences against each target gene and controls (supplemental Table 3) was cloned into the vector pLT3GGmirEPPIR [26] (supplemental Table 4). Screens were performed as previously described as negative selection screens [27]. U266-N1ΔE and empty vector control cells (U266-vector) were transduced with the pooled library. After doxycycline induction, cells were treated with DMSO or Len (1 µM), Mel (1.25 µM), or Btz (0.8 nM). Changes in shRNA frequencies were detected by next-generation sequencing using an NextSeq 500 system (Illumina) and bioinformatic analyses [28]. 

### Chromatin immunoprecipitation with high-throughput sequencing (ChIP-seq)

Cells were crosslinked with 1% formaldehyde and then treated with micrococcal nuclease (New England Biolabs). Cells were lysed in ChIP-RIPA buffer (10 mM Tris–HCl pH 7.5, 150 mM NaCl, 1% NP-40, 1% DOC, 0.1% SDS, 1 mM EDTA) and sonicated with a Branson sonifier to obtain DNA fragments shorter than 1 kb. Chromatin was pre-cleared with Protein A-Sepharose beads (New England Biolabs) and immunoprecipitated with RBPJ (RBPSUH, D10A4, Cell Signaling Technology) XP rabbit monoclonal antibody. Chromatin/protein complexes were collected by incubation with Protein A magnetic beads (New England Biolabs) for 6 h. After several washings, chromatin was eluted with 1% SDS/0.1 M NaHCO_3_ and crosslinks were reversed overnight at 65 °C. DNA was purified by phenol-chloroform extraction. Libraries for ChIP-seq were constructed using the NEBNext Ultra DNA Library Prep Kit for Illumina (New England Biolabs). Briefly, ChIP DNA was end-repaired, A-tailed, Illumina adaptors were ligated, and the DNA was separated on a 2% agarose gel. DNA fragments of 200 bps were excised and purified using a Monarch DNA Gel Extraction Kit (New England Biolabs). Size-selected DNA was amplified with NEBNext Multiplex Oligos for Illumina (Index Primer Set 1 and Set 2) by PCR, purified with Agencourt AMPure XP Beads (Beckman Coulter) and the concentration of the libraries was estimated with the Quanti-iTTM PicoGreen dsDNA Assay Kit (Thermo Fisher Scientific). Sequencing of the DNA libraries was performed on a NextSeq 500 System (Illumina).

### Cell viability and proliferation assays

Cell viability was determined with the CellTiter-Glo^®^ assay (Promega, Fitchburg, WI, USA) and a Luminoskan Ascent Microplate Luminometer (Thermo Fisher Scientific). For the proliferation assays, cells were counted over 15 days using trypan blue solution (Sigma-Aldrich). Subsequently, cells were re-seeded at the initial density, and fresh drugs were added to the culture medium.

### Antibodies

All antibodies (supplemental Table 5) were used according to their protocol for flow cytometry analysis on a BD FACSCanto™ II (BD Biosciences, Milpitas, CA, USA) or for immunoblotting [29]. 

### Quantitative PCR (qPCR) analysis

Total RNA was extracted with the NucleoSpin^→^ RNA Mini kit (Macherey-Nagel, Düren, Germany) and qPCR was performed (supplemental Table 6) as described before [30], starting with 5 µg RNA for each reverse transcription reaction using SuperScript™ III Reverse Transcriptase (Thermo Fisher Scientific). For qPCR, the SYBR™ Select Master Mix for CFX (Thermo Fisher Scientific) and a StepOnePlus™ Real-Time PCR System (Applied Biosystems, Waltham, MA, USA) were used. Hypoxanthine-guanine phosphoribosytransferase (*HPRT*) or glyceraldehyde 3-phosphate dehydrogenase (*GAPDH*) served as housekeeping gene. Log_2_ fold changes (log_2_FC) were calculated with the ΔΔCt-method using the StepOne-Plus Software (Applied Biosystems).

### Immunoblotting

Immunoblotting was performed as previously described [31]. Briefly, cells were lysed using NP-40 buffer (50 mM Tris pH 7.5, 150 mM NaCl, 1% Triton, 0.5% NP40, and 0.2% SDS) and protein concentrations were determined with Roti^®^-Quant reagent (Carl Roth GmbH, Karlsruhe, Germany). For each sample, 50 µg protein was mixed with Laemmli buffer, boiled, and separated through 10% SDS-PAGE. Subsequently, proteins were transferred onto a Protran^®^ nitrocellulose membrane (Whatman, Dassel, Germany). Membranes were blocked with 5% skim milk in TBS-T buffer (50 mM Tris, 150 mM NaCl, and 0.1% Tween-20) and probed with specific primary and secondary antibodies (supplemental Table 5) overnight at 4 °C and for 60 min at room temperature, respectively. Histone H3 served as loading control. Proteins were detected using SuperSignal™ West Femto Maximum Sensitivity Substrate (Thermo Fisher Scientific), SuperSignal™ West Pico PLUS Chemiluminescent Substrate (Thermo Fisher Scientific), and a ChemiDoc MP Imaging System (Bio-Rad Laboratories, Hercules, CA, USA). Quantification was performed using Image Lab software (Bio-Rad Laboratories).

### Reporter assays

Cells were seeded at a density of 6 × 10^4^ cells/well in 500 µL antibiotic free RPMI medium, 14 h prior to transfection. In each well, 2.5 µg of *NFIL3* promoter reporter vector (kindly provided by René A. A. St.-Arnaud [32]) and 250 ng of Renilla luciferase internal control vector (pTK-Green Renilla Luc Vector, Thermo Fisher Scientific) were transfected using the Lipofectamine 3000 Reagent (Invitrogen, Carlsbad, CA, USA) following the manufacturer’s instructions. 30 h post-transfection, the Dual-Luciferase^®^ Reporter Assay (Promega) was performed according to the manufacturer’s protocol. Results were calculated by normalizing the relative light units of Firefly luciferase to Renilla luciferase after subtracting the background signal and are expressed as fold change over the empty vector.

### Animals

Animal experiments were carried out according to the replacement, reduction, refinement (3Rs) principle. All experiments were carried out as approved by the local authorities (RUF55.2.2-2532-2-1204-22, Regierung Unterfranken, Würzburg, Germany). For MM induction, 2.5 × 10^4^ MOPC.315.BM.Luc.eGFP-*mCrbn*^*I391V*^ cells were intratibially injected into female BALB/cAnCrl mice, obtained from Charles River Laboratories (Sulzfeld, Germany) at seven weeks of age and after acclimatization for ten days. The *mCrbn*^*I391V*^-expressing, Len-sensitive MOPC.315.BM cell line was kindly provided by Jan Krönke. On day 7, mice were randomized into four groups prior to treatment over 19 days with [1] i.p. injection of vehicle (25 mg/kg polymer; *n* = 10) [2], i.p. injection of RA (3 mg/kg; *n* = 10) [3], i.p. injection of Len (5 mg/kg; *n* = 10) [4], i.p. injection of RA (3 mg/kg) and Len (5 mg/kg) (*n* = 12). Tumor load was determined by BLI imaging [33]. Mice with severe signs of EMD were sacrificed under the criteria of human endpoints of the animal study protocol. The intervention ended on day 26 after i.t. injection. Injected tibiae were extracted, fixed with % PFA for 24 h, and stored in 7% ethanol. For microCT analysis to determine bone destruction, the fixed bones were scanned on the Skyscan 1172 v1.5 (Kontich, Belgium) according to the guidelines of the American Society for Bone and Mineral Research [34]. After microCT scanning, the bones were decalcified in EDTA for 14 days and embedded into paraffin. For Tartrate-resistant acid phosphatase (TRAP) staining, 4 μm longitudinal sections were prepared and stained for 2 h in TRAP staining solution (100 ml TRAP buffer + 1 ml TRAP solution, adjustment of pH to 5.0, addition of 0.06 g Fast Red Violet LB Salt. TRAP buffer: 0.33 g sodium acetate + 0.23 g Di-sodium tartrate dehydrate + 100 ml Aqua destillata + 0.01 g Naphthol phosphate disodium salt + 1 ml Dimethylformamide). Osteoclasts were counted in a 250 × 450 μm rectangular ROI below the metaphysis. Osteoclasts were counted on trabecular and cortical bone and were identified based on positive TRAP staining, more than one nucleus, and location directly on the bone surface. The osteomeasure system (OsteoMetrics, Decatur, GA, USA) was used for osteoclast analysis.

### Drug-loaded micellar formulations for in vivo administration of drugs

The biocompatible triblock copolymer PMeOx_35_-*b*-PBuOzi_20_-*b*-PMeOx_35_ (A-pBuOzi-A), where MeOx is poly(2-methyl-2-oxazoline) and BuOzi is poly(2-n-butyl-2-oxazine), and thin film hydration method were used to prepare drug-loaded micelles from methanol stock solutions, as previously described [35, 36]. Lyophilized samples were reconstituted freshly and filtered through 0.2 μm syringe filters prior to injection in mice.

### Statistical analysis

For statistical analyses Prism 9 (GraphPad, Boston, MA, USA) was used, including Spearman’s correlation analysis, student’s *t*-test, and one or two-way ANOVA. An unpaired, non-parametric Mann-Whitney-U test was performed with Prism 11.0.1 (GraphPad) to analyze whether patients with high NFIL3 mRNA or high MYC protein expression [37] are enriched in patient groups with a high mutational count or extramedullary disease.

For gene expression stratification we used the gene of interest (NFIL3) and samples were stratified into three groups based on their expression:

- High expression: TPM > 90th percentile.
- Low expression: TPM < 10th percentile.
- Intermediate expression: TPM between the 10th and 90th percentiles.

Kaplan–Meier survival curves and their corresponding significant testing were generated using the survival package in R [38]. Differences between groups were evaluated using the log-rank test. For each gene, survival was compared between high and low expression groups, excluding intermediate samples. Cox proportional hazards regression models were fitted to estimate hazard ratios (HRs) and 95% confidence intervals (CIs). P-values were adjusted using the BH method [39]. 

Cytogenetic abnormalities such as del(17p), p53 mutations, gain/amp(1q), t(4;14), t(14;16), t(14;20) were identified as previously described [22]. We used the Wilcoxon rank-sum tests with Benjamini-Hochberg correction [39] to compare the gene expression between cytogenetically defined groups (presence versus absence of abnormality), for each of the genes of interest. Survival analyses with their corresponding multivariate Cox regression models were also performed within subsets of patients stratified by both gene expression and cytogenetic status.

## Results

### Transcriptome profiling of Notch-depleted MM cells identifies novel Notch1 effectors

**Fig. 1:**
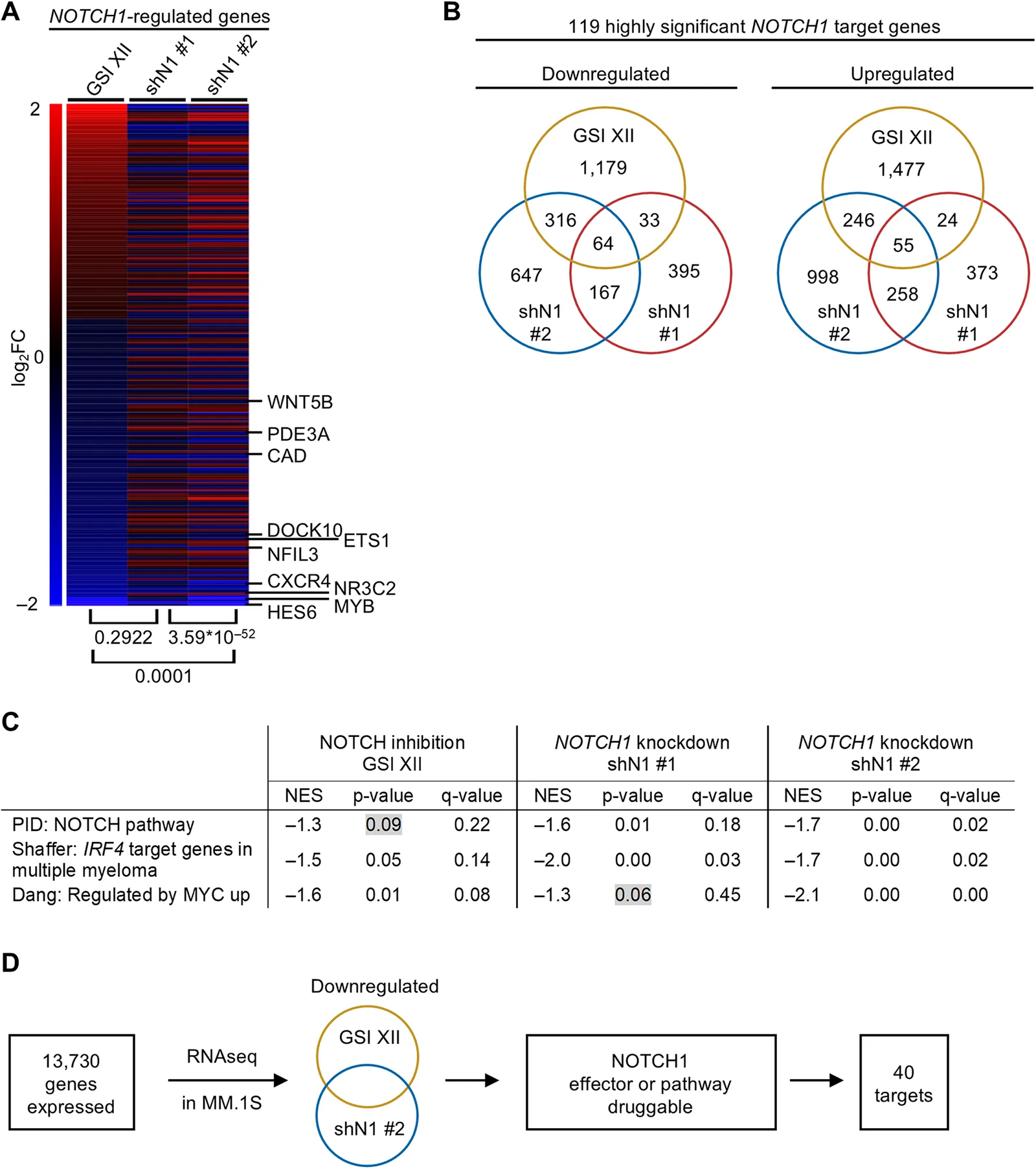
Transcriptome profiling of Notch1-depleted MM.1S cells identifies novel downstream effectors. (**A**) Heatmap comparing the direction of regulation of the 119 highly significantly differentially expressed genes under conditions of Notch inhibition (GSI XII) and *NOTCH1* knockdown (shN1 #1, shN1 #2). Genes were ordered according to the log_2_FC in the GSI XII conditions. *P*-values for correlation were calculated using Spearman’s rank test. Ten out of 40 genes included in the targeted shRNA library were significantly downregulated under all three conditions (GSI XII, shN1 #1, shN1 #2) and the position is marked next to the heatmap. (**B**) Venn diagrams depicting the overlap of significantly *NOTCH1* down- and upregulated genes in MM.1 S cells treated as indicated (*padj* < 0.05). (**C**) Flow chart showing the selection process of druggable Notch1 targets or pathways after *NOTCH1* knockdown (shN1 #1, shN1 #2 versus empty vector) or inhibition of the Notch pathway by 5 µM GSI XII versus DMSO in MM.1S cells. Of 13,730 expressed genes (counts per million > 1), significantly downregulated targets were selected as candidates (*padj* < 0.05, log_2_FC < 0). These genes were further filtered for druggability. (**D**) Panel shows the GSEA of genes downregulated in response to Notch inhibition (GSI XII) and after *NOTCH1* knockdown (shN1 #1 and shN1 #2). Normalized enrichment scores (NES) and q-values of three gene sets are shown including the Notch pathway gene set (PID, pathway interaction database), the IRF4 target gene set in MM (Shaffer [42]), and the gene set regulated by MYC up (Dang [43]). Light gray shading highlights non-significant *p*-values (> 0.05)

To identify Notch1 downstream effectors in MM cells [8], we performed RNAseq analysis of MM.1S cells after blocking signaling activation with GSI XII or shRNA-based *NOTCH1* knockdown (shN1 #1; shN1 #2; Fig. 1A). MM.1S cells express all four Notch receptor isoforms [8]. GSI XII acts as a competitive inhibitor of the γ-secretase complex and blocks the cleavage and activation of all Notch isoforms on the membrane [40]. This approach was combined with the specific knockdown of only *NOTCH1* to inhibit signal transduction using both a pharmacological and a genetic approach. Notch1 depletion decreased the expression of the Notch1 target genes *HEY1*, *HES1*, and *HES6* in MM.1S cells, confirming inhibition of the pathway using both approaches (supplemental Fig. 1). Transcriptome profiling revealed that under all three conditions, many of the 13,730 expressed genes were up- and downregulated (GSI XII: 1,802 up and 1,592 down; shN1 #1: 710 up and 659 down; shN1 #2: 1,557 up and 1,194 down; cut-off: *padj* < 0.05, Fig. 1B). Direct comparisons indicated that *NOTCH1* knockdown had similar effects on gene expression with both shRNAs (shN1 #1, shN1 #2; *r* = 0.79, *P* < 3.59 × 10^− 52^). Differences in numbers of differentially expressed genes may arise from shRNAs targeting different sequences on the target gene resulting in different knockdown efficiencies. Furthermore, the noise in the dataset effects the number of genes that can be called significantly regulated by a bioinformatic algorithm such as edgeR. In addition, GSI XII treatment showed no significant correlation with shN1 #1 (*r* = 0.069, *P* < 0.3) and a weak correlation with shN1 #2 (*r* = 0.25, *P* < 0.0001; Fig. 1A). This could be explained by known off-target effects of GSI XII and the fact that the gamma-secretase complex has many other substrates besides Notch [41]. Genes that were concomitantly downregulated under all three conditions are indicated based on their position in a heatmap (Fig. 1A). Gene set enrichment analysis (GSEA) revealed that Notch depletion reverted the expression of a Notch-related gene signature, an interferon regulatory factor 4 (IRF4) target gene set previously identified in MM [42] and a MYC target gene signature [43], confirming that MYC is a Notch target in MM (Fig. 1C), as previously described [44]. Since shN1 #2 and GSI XII treatment showed a higher correlation, we compared all significantly downregulated genes between these two conditions and further evaluated the overlapping genes for relevance to MM pathogenesis and druggability based on literature search (Fig. 1D). 40 genes were selected as potential targets (supplemental Table 2).

### Focused genomics screens reveal NFIL3 as a druggable target mediating resistance of MM cells

**Fig. 2:**
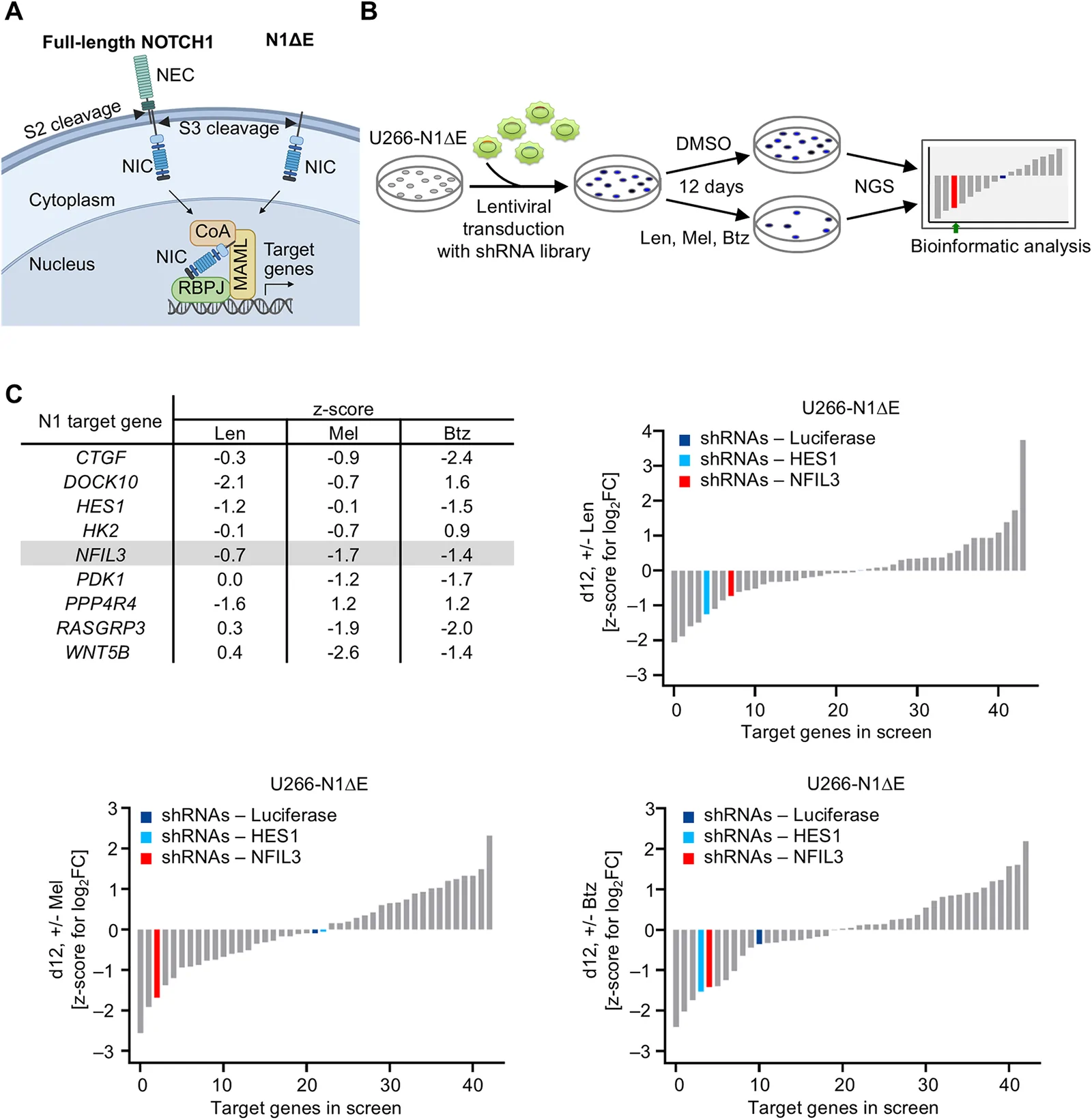
Focused shRNA library screens identify nine novel druggable Notch1 target genes. (**A**) Scheme of Notch1 signaling in U266-N1∆E cells compared to wildtype Notch1 signaling. (**B**) Scheme for the focused shRNA screens performed in U266-N1ΔE cells. After transduction of the shRNA library, cells were treated with DMSO or with Len (1 µM), Mel (1.25 µM), or Btz (0.8 nM) for 12 days. ShRNA sequences were recovered and changes in shRNA frequencies were detected using NGS. Bioinformatic analyses, including the calculation of z-scores, were performed. (**C**) Results of the focused shRNA screens in U266-N1ΔE cells. Z-scores of nine genes are shown that can serve as druggable targets based on literature search. Waterfall plots showing z-scores for log_2_FC in shRNA frequencies (median of five different shRNAs) between Len, Mel, Btz, and DMSO-treated cell populations after 12 days of growth. Panels show the median z-score for shRNAs targeting genes as indicated

To determine whether the 40 Notch1 target genes mediate drug resistance in MM cells, we generated a focused shRNA library containing five shRNAs per gene. Representation of individual shRNAs in the plasmid pool was determined by next-generation sequencing (NGS; supplemental Fig. 2). The screens were carried out in U266 cells with low endogenous Notch1 levels versus engineered U266 cells expressing the Notch1 variant N1ΔE (supplemental Fig. 3), a Notch1 variant that consists of the intracellular active domain and lacks the extracellular domain, allowing ligand-independent cleavage and constitutive activation of Notch1 (Fig. 2A). After transduction of the shRNA library, the cells were incubated with Len, Mel, and Btz for 12 days (Fig. 2B). Bioinformatic analyses were then performed to identify depleted shRNAs in the remaining cell populations under drug treatment compared with DMSO-treated controls (Fig. 2B). Screens identified nine druggable hits including CTGF, HES1, and WNT5B that exhibited a significant depletion of shRNAs based on z-scores for log_2_FC under at least two conditions (z-score < -0.5, Fig. 2C, supplemental Table 7). Only NFIL3 showed a significant depletion under all three conditions and was therefore chosen for validation in a downstream study (Fig. 2C, marked in red).

### ChIP-seq analysis and reporter assays prove NFIL3 as a downstream target of Notch1/RBPJ signaling

**Fig. 3:**
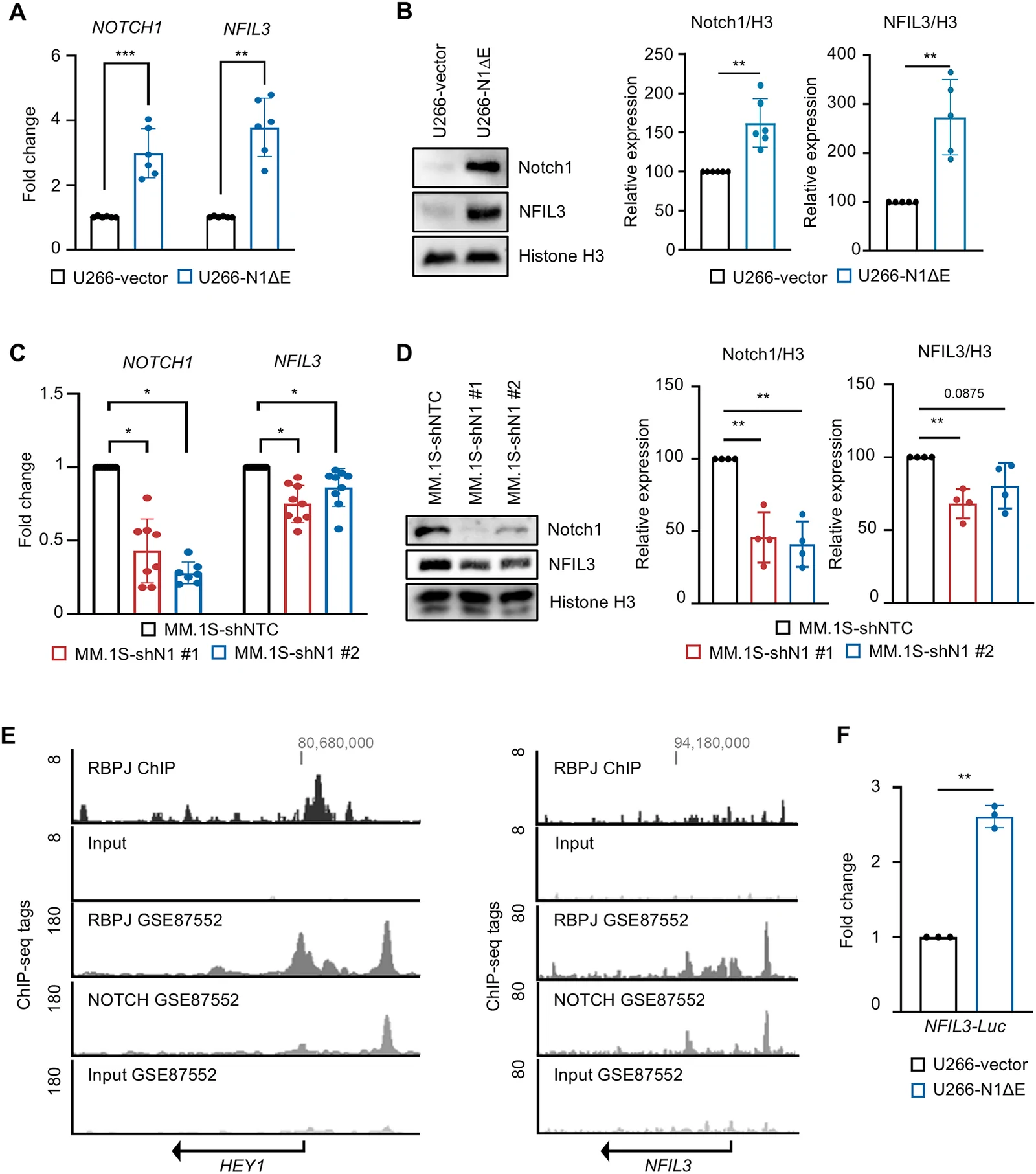
ChIP-seq analysis and reporter assays reveal NFIL3 as a downstream target of the Notch1/RBPJ signaling. (**A**) Quantitative PCR analysis of *NOTCH1* and *NFIL3* in U266 cells expressing the empty vector (U266-vector) or the Notch1 variant N1ΔE (U266-N1ΔE). (**B**) Immunoblots of whole extracts of U266 cells. Blots were probed with antibodies against Notch1, NFIL3 and Histone H3. Data are shown as mean ± SD of six biological replicates. (**C**) Quantitative PCR analysis of *NOTCH1* and *NFIL3* in MM.1S cells after *NOTCH1* knockdown (shN1 #1, shN1#2). Data are shown as mean ± SD of eight or nine biological replicates. (**D**) Blots were probed with antibodies against Notch1, NFIL3 and Histone H3. Data are shown as mean ± SD of four biological replicates. Paired *t*-tests were used for statistical analysis. *P-value ≤ 0.05, **P-value ≤ 0.01, ***P-value ≤ 0.001. (**E**) Integrated genome browser tracks showing the signal for RBPJ binding and input DNA at the *HEY1* and *NFIL3* loci. Upper panels show ChIP-seq analysis of MM.1S cells using RBPJ antibodies (this study). Lower panels show the analysis of published ChIP-seq data with Notch1 and RBPJ antibodies (GSE87552). (**F**) U266-vector and U266-N1ΔE cells were transfected with the *Nfil3* promoter reporter vector (− 818/+182 bp) and luciferase activity was measured

To show that NFIL3 is a Notch1 target gene, we analyzed two cell lines, one MM cell line with Notch1 expression (MM.1S) and one with low to undetectable Notch1 expression (U266). We expressed the constitutively active Notch1 form N1ΔE in U266 cells and observed a significant increase in NFIL3 expression both at transcriptional and translational levels (Fig. 3A, B). Additionally, shRNA mediated knockdown of Notch1 in MM.1S cells led to a downregulation of NFIL3 RNA and protein levels (Fig. 3C, D). We confirmed the result of our genomic screens and showed that NFIL3 is a novel downstream target of Notch1. To confirm that NFIL3 is a transcriptional target of the canonical Notch1/RBPJ signaling complex, we performed ChIP-seq analysis in MM.1S cells using antibodies against RBPJ, which is a key DNA-binding transcription factor and co-activator of the Notch pathway (Fig. 3E). In parallel, analysis of published ChIP-seq data in human umbilical vein endothelial cells demonstrated that Notch1/N1IC occupies genomic regulatory regions in association with the DNA-binding factor RBPJ [45]. The results indicated the prominent recruitment of RBPJ to known Notch target genes including *HEY1*. RBPJ was also associated, albeit weakly, with the *NFIL3* locus in regions upstream and around the transcription start site as well as intronic sequences. In addition, we performed a reporter assay for NFIL3 in U266 cells after upregulation of Notch1 (U266-N1ΔE), showing that the transcriptional activity of the NFIL3 reporter construct is positively regulated by N1ΔE expression, further confirming that NFIL3 is a transcriptional target of canonical Notch1/RPBJ signaling (Fig. 3F).

### Knockdown of NFIL3 targets the MYC axis and affects viability and growth of MM cells

**Fig. 4:**
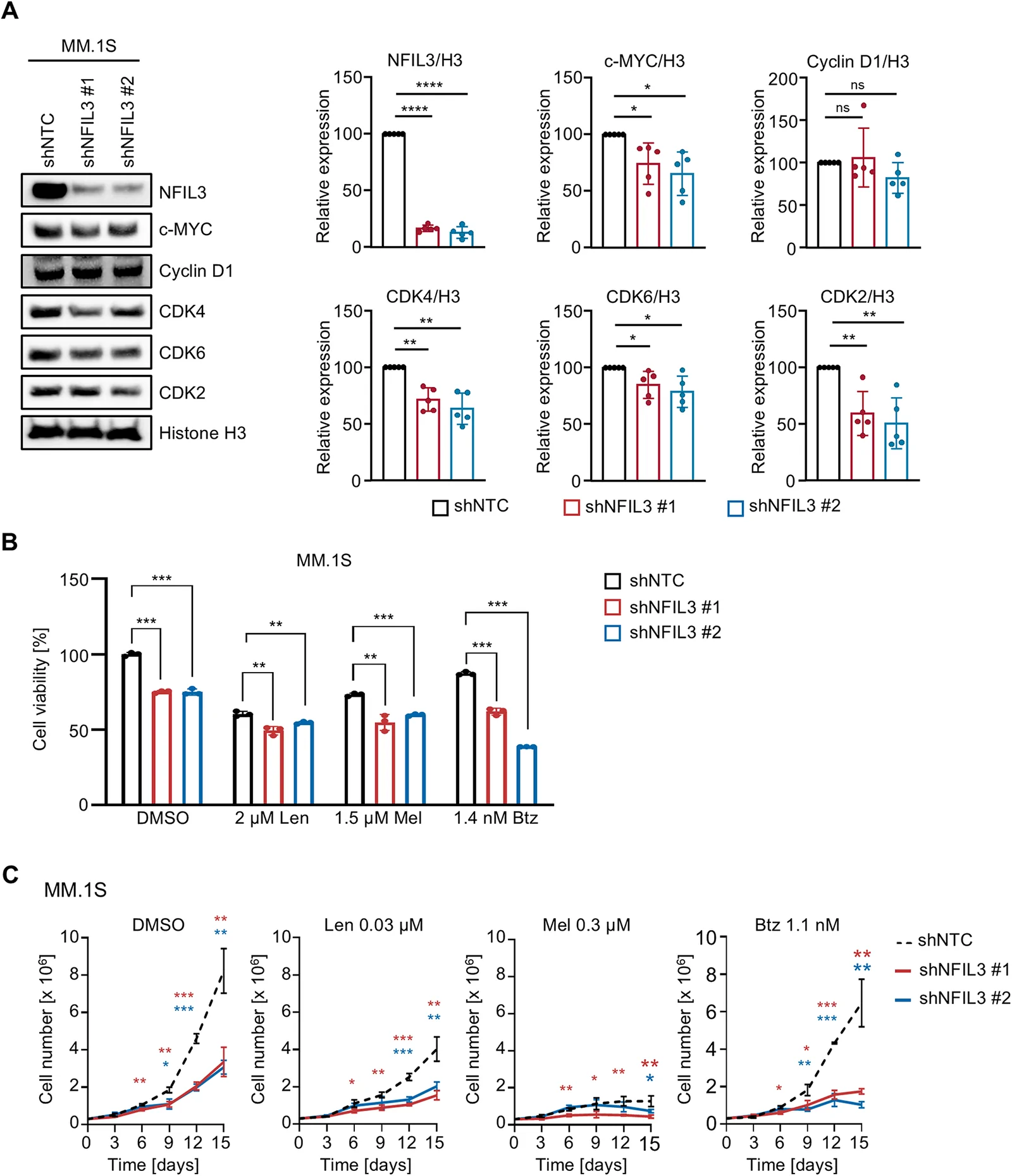
Knockdown of *NFIL3* controls a MYC-driven transcriptional program, viability, and growth of MM.1S cells.** (A**) Immunoblots of whole extracts of MM.1S cells after *NFIL3* knockdown. Blots were probed with antibodies against NFIL3, c-MYC, Cyclin D1, CDK4, CDK6, CDK2, and Histone H3. Quantification was performed for three to five biological replicates using Image Lab. Values were normalized to shNTC and were set to 100. Paired *t*-tests were used for statistical analysis. *P-value ≤ 0.05, **P-value ≤ 0.01, ****P-value ≤ 0.0001. (**B**) Cell viability of human MM.1S cells after knockdown of *NFIL3* (shNFIL3 #1, shNFIL3 #2) and treatment with Len, Mel, and Btz for three days at the indicated concentrations was determined using the CellTiter-Glo^®^ Luminescent Cell Viability Assay. Data are shown as mean ± SD of three biological replicates (*n* ≥ 3). Unpaired t-tests were used for statistical analysis. (**C**) Cell counts of MM.1S cells after *NFIL3* knockdown grown over 15 days in media containing DMSO, Len, Mel, and Btz at the indicated concentrations. Representative results are shown for one out of three independent experiments and are given as the mean ± SD. Unpaired *t*-tests were used for statistical analysis. *P-value ≤ 0.05, **P-value ≤ 0.01, ***P-value ≤ 0.001, ****P-value ≤ 0.0001

To evaluate the transcriptional and tumor biological effects of NFIL3 depletion, we generated a shRNA mediated knockdown of NFIL3 in MM.1S cells (shNFIL3 #1, shNFIL3 #2). Immunoblotting revealed that NFIL3-depleted MM.1S cells had lower c-MYC and cyclin-dependent kinase (CDK) levels of CDK4, CDK6, and CDK2, indicating c-MYC as a downstream target of NFIL3 (Fig. 4A). Additionally, we generated a shRNA mediated knockdown of NFIL3 in Notch1 overexpressing U266-N1ΔE cells and demonstrated a decrease in c-MYC levels despite Notch1 overexpression (supplemental Fig. 4). Furthermore, low NFIL3 levels correlated with lower levels of Stat3, pStat3 (Y705), and pStat3 (S727) (supplemental Fig. 4). Moreover, knockdown of NFIL3 decreased cell viability and growth of MM.1S cells and slightly increased the cytotoxic effect of Len, Mel, and Btz (Fig. 4B, C), also in the MM cell lines RPMI 8226 and LP-1 (Supplemental Fig. 5). These data indicate that NFIL3 positively regulates the expression of c-MYC and CDKs, which consequently leads to decreased viability and growth of MM.1S cells lacking NFIL3.

### High expression of NFIL3 is associated with inferior survival, high-risk cytogenetic abnormalities, and higher incidence of extramedullary disease at biopsy

**Fig. 5:**
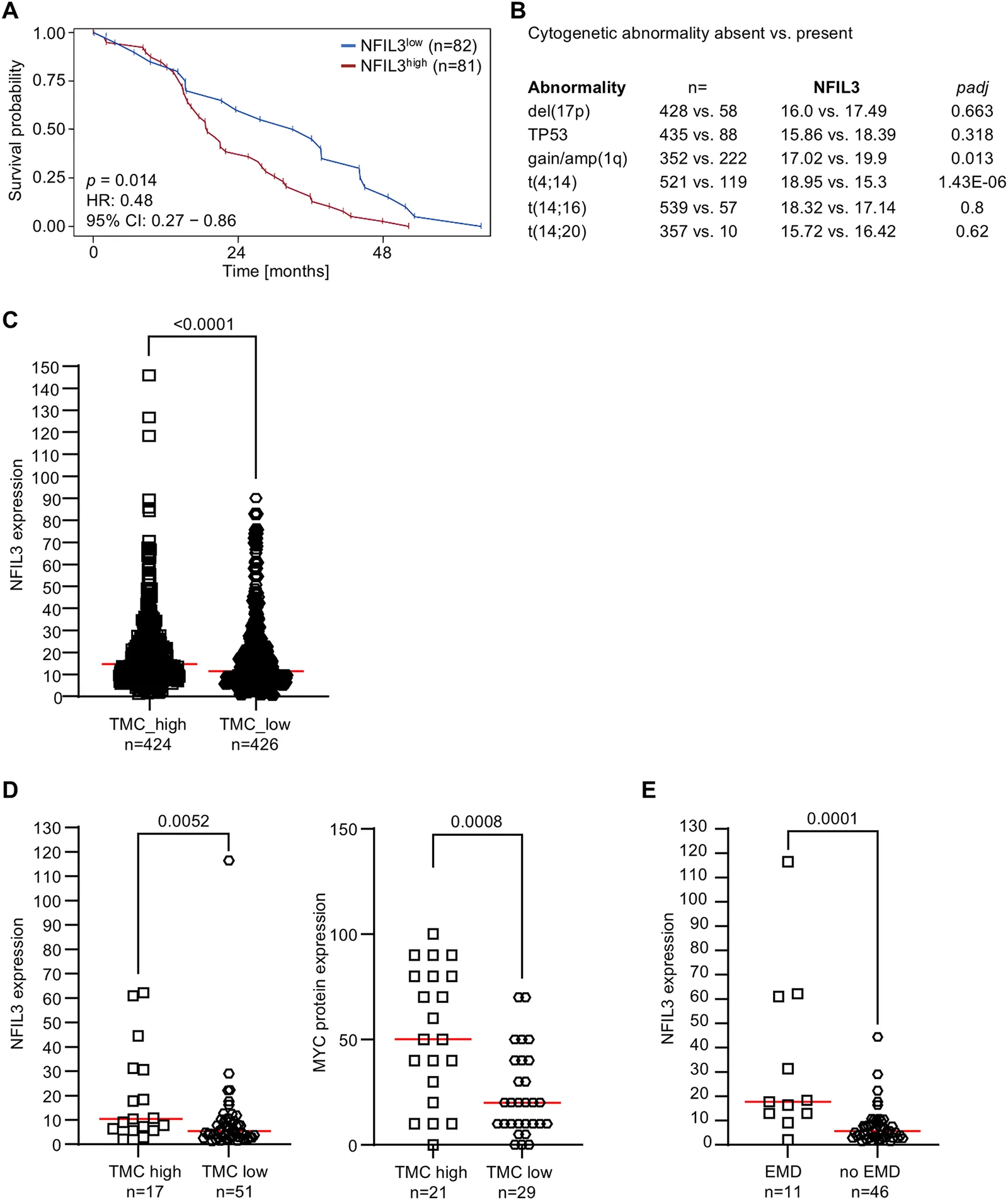
High NFIL3 expression levels are an independent prognostic marker in MM. (**A**) Survival analysis using a Kaplan-Meier plot and assessing the effect of high NFIL3 on overall survival in the MMRF cohort. Plot demonstrates probability for prolonged survival if NFIL3 gene expression is low (blue line, *p* = 0.014, Cox single variable regression model) compared to high levels (red line). (**B**) Comparison of gene expression of NFIL3 between samples with and without cytogenetic abnormalities associated with a high-risk status. Statistical test was Mann-Whitney-U. *P*-values were adjusted (*padj*) for testing of multiple hypotheses using the Benjamini-Hochberg procedure. The column labeled with “NFIL3” depicts the TPM counts for the NFIL3 gene, “abnormality present” versus “no abnormality”. (**C**) Comparison of gene expression of NFIL3 between unpaired samples from patients with higher and lower tumor mutational count (TMC) in the MMRF cohort using unpaired Mann-Whitney-U test. (**D**) Comparison of gene expression of NFIL3 and c-MYC levels between unpaired samples from patients with higher and lower TMC in the validation cohort using unpaired Mann-Whitney-U test. (**E**) Comparison of gene expression of NFIL3 between unpaired samples from patients with or without EMD (Yes: *n* = 11 or No: *n* = 46) at biopsy in the validation cohort using an unpaired Mann-Whitney-U test

To determine the prognostic potential of high NFIL3 expression, we compared gene expression levels of NFIL3 with clinical parameters in two patient cohorts, the MMRF cohort (MMRF CoMMpass study cohort) and a published validation cohort [22]. Higher expression of NFIL3 was associated with shorter overall survival (Fig. 5A), and a high incidence of the high-risk cytogenetics gain/amp(1q) (Fig. 5B) in the MMRF cohort. We fitted a multivariate cox regression model to test for significant survival considering NFIL3 levels and gain/amp(1q), revealing that high NFIL3 gene expression is significantly associated with lower survival in the MMRF cohort. Furthermore, a higher tumor mutational count correlated with higher NFIL3 expression in the MMRF cohort (Fig. 5C). In the validation cohort, we observed a higher tumor mutational count in patient samples with higher NFIL3 mRNA expression and c-MYC protein expression (Fig. 5D). In addition, we detected a higher incidence of EMD at biopsy in patients with higher NFIL3 expression (Fig. 5E). These data provide evidence that high expression of NFIL3 is associated with high-risk cytogenetics and progressive disease.

### RA blocks the NFIL3-MYC axis, induces apoptosis, and reduces viability and growth of MM.1S cells

**Fig. 6:**
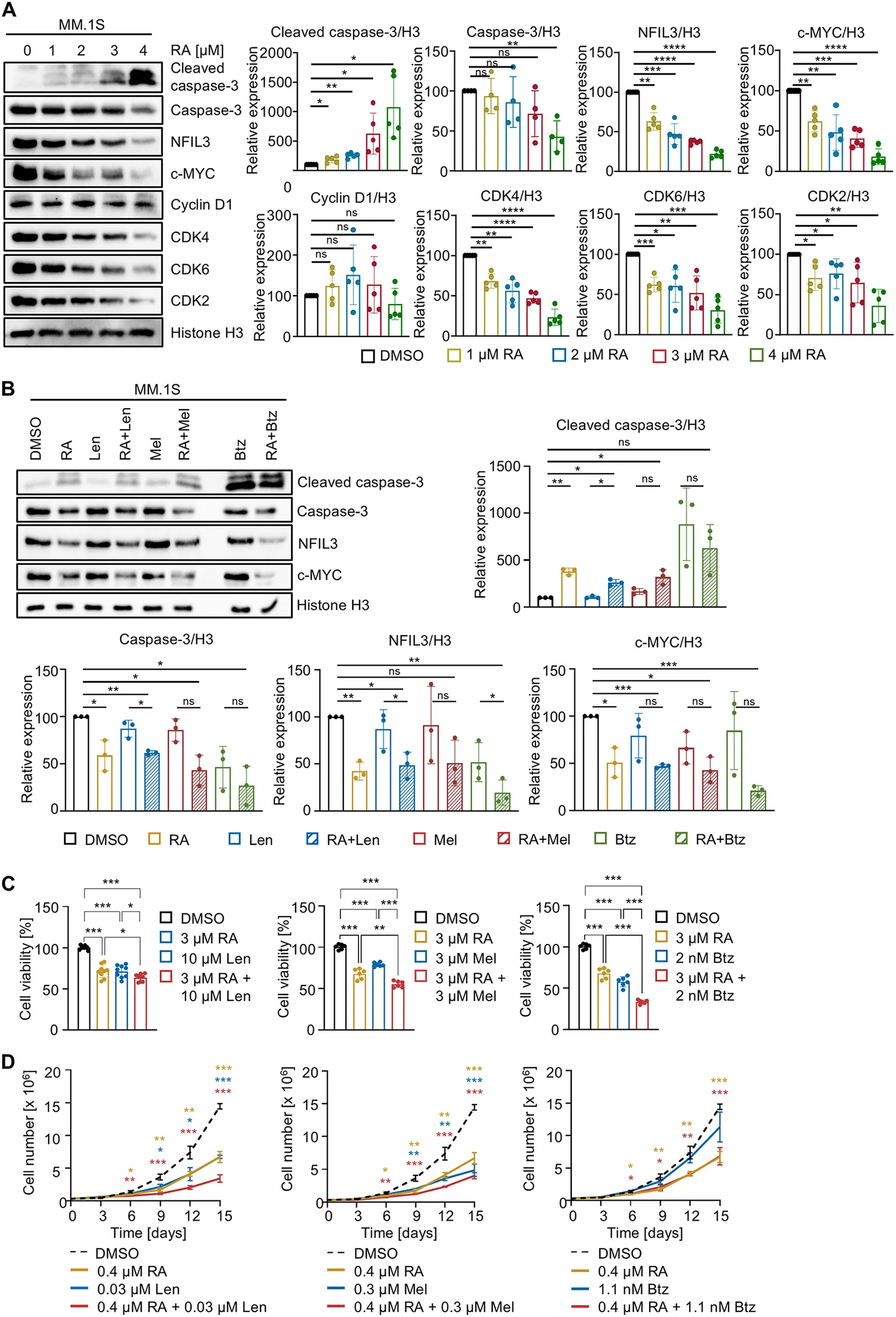
RA targets the NFIL3-MYC axis and controls viability and growth of MM.1S cells. **(A**) Immunoblots of whole extracts of MM.1S cells after treatment with RA for 24 h. Blots were probed with antibodies against cleaved caspase-3, caspase-3, NFIL3, c-MYC, Cyclin D1, CDK4, CDK6, CDK2, and Histone H3. Paired *t*-tests were used for statistical analysis. *P-value ≤ 0.05, **P-value ≤ 0.01, ***P-value ≤ 0.001, ****P-value ≤ 0.0001. **(B**) Immunoblots with whole extracts of MM.1S cells after treatment with RA, Len, Mel, Btz for 24 h. Blots were probed with antibodies against cleaved caspase-3, caspase-3, NFIL3, c-MYC, and Histone H3. Quantification was performed for three to five biological replicates using Image Lab. Paired *t*-tests were used for statistical analysis. *P-value ≤ 0.05, **P-value ≤ 0.01, ***P-value ≤ 0.001. (**C**) Cell viability of MM.1S cells treated with RA, Len, Mel, and Btz alone or in combination for 48 h at the indicated concentrations. Data are presented as the mean ± SD of three independent experiments, each dot representing one replicate. Unpaired *t*-tests were used for statistical analysis. (**D**) Cell counts of MM.1S cells grown over 15 days in media containing DMSO, RA, Len, Mel, and Btz alone or in combination at the indicated concentrations. The three line graphs share the same DMSO and RA datasets. Data are presented as the mean ± SD of one representative experiment from at least three independent experiments. Unpaired *t*-tests were used for statistical analysis. *P-value ≤ 0.05, **P-value ≤ 0.01, ***P-value ≤ 0.001, ****P-value ≤ 0.0001

To pharmacologically target NFIL3, we used the oleanane-type triterpenoid saponin RA as it has been used to overcome the STAT3-NFIL3 axis mediated drug resistance in choriocarcinoma cells [46]. Increasing doses of RA suppressed viability of the human MM cell lines MM.1S, OPM-2, LP-1, IM-9, AMO-1, RPMI 8226, U266-vector, U266-N1ΔE, and the murine cell line MOPC.315.BM.Luc.eGFP-*mCrbn*^*I391V*^ (Supplemental Fig. 6). RA induced cleaved caspase-3 activity and downregulated the expression of NFIL3, STAT3, pSTAT3 (S727), c-MYC, CDK4, CDK6 and CDK2 in MM.1S cells (Fig. 6A, supplemental Fig. 4). In parallel, RA slightly enhanced the efficacy of Len and Mel, as evidenced by increased levels of cleaved caspase-3 (Fig. 6B). The increase in cleaved caspase-3 levels was significant for treatment with RA plus Len, and a similar trend was observed for RA plus Mel. However, we did not observe an increase in MM cells treated with RA plus Btz, possibly because Btz alone already exerts a strong cytotoxic effect. Furthermore, RA decreased levels of NFIL3 and c-MYC in combination with the drugs (Fig. 6B). RA treatment reduced viability (Fig. 6C) and growth (Fig. 6D) either alone or in combination with Len, Mel, and Btz.

### RA blocks the NFIL3-MYC axis in MOPC.315.BM.Luc cells and RA nanocarriers control tumor growth and osteolytic bone disease in MM mice

**Fig. 7:**
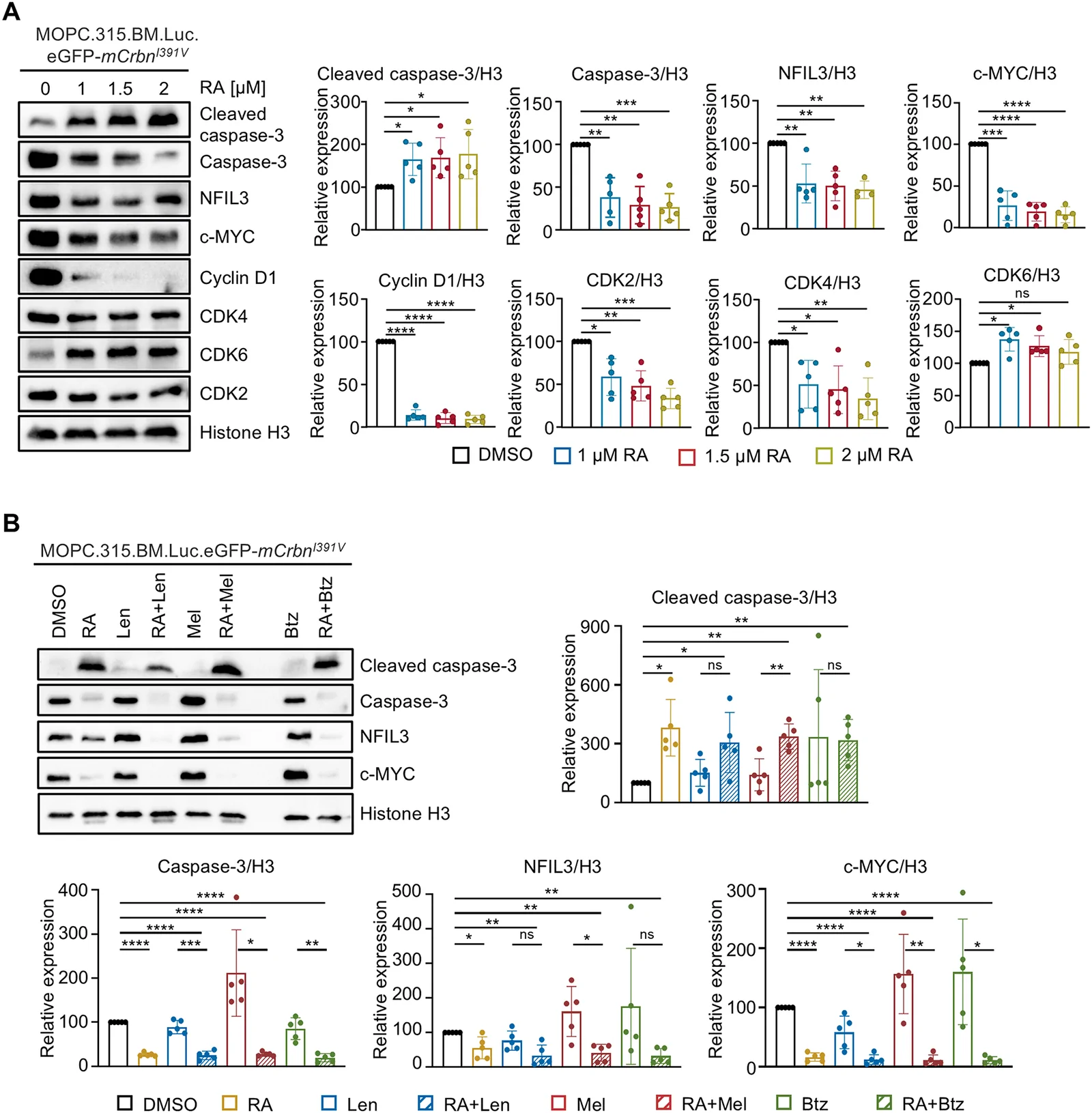
RA targets the NFIL3-MYC axis in MOPC.315.BM.Luc.eGFP-*mCrbnI*^*391 V*^ cells. (**A**) Immunoblots of whole extracts of MOPC.315.BM.Luc.eGFP-*mCrbn*^*I391V*^ cells after treatment with RA for 24 h. Blots were probed with antibodies against cleaved caspase-3, caspase-3, NFIL3, c-MYC, Cyclin D1, CDK4, CDK6, CDK2, and Histone H3. (**B**) Immunoblots with whole extracts of MOPC.315.BM.Luc.eGFP-*mCrbn*^*I391V*^ cells after treatment with RA, Len, Mel, Btz for 24 h. Blots were probed with antibodies against cleaved caspase-3, caspase-3, NFIL3, c-MYC and Histone H3. Quantification was performed for three to five biological replicates using Image Lab. Paired *t*-tests were used for statistical analysis. *P-value ≤ 0.05, **P-value ≤ 0.01, ***P-value ≤ 0.001, ****P-value ≤ 0.0001

Consistently, RA reduced cell viability of murine MOPC.315.BM.Luc.eGFP-*mCrbn*^*I391V*^ cells with increasing doses (supplemental Fig. 6), induced cleaved caspase-3 activity and downregulated the NFIL3-MYC axis as evidenced by immunoblotting (Fig. 7A, supplemental Fig. 4). The addition of RA to Mel enhanced apoptosis and downregulated the NFIL3-MYC axis in MOPC.315.BM.Luc.eGFP-*mCrbn*^*I391V*^ cells. Addition of RA to Len also showed a trend towards increased cleaved caspase-3 levels, however, this was not significant and an increase in cleaved caspase-3 levels was not observed for the samples where RA was added to Btz in MOPC.315.BM.Luc.eGFP-*mCrbn*^*I391V*^ cells (Fig. 7B). In parallel, RA decreased NFIL3 and c-MYC protein levels either alone or in combination with Len, Mel, and Btz (Fig. 7B).

**Fig. 8:**
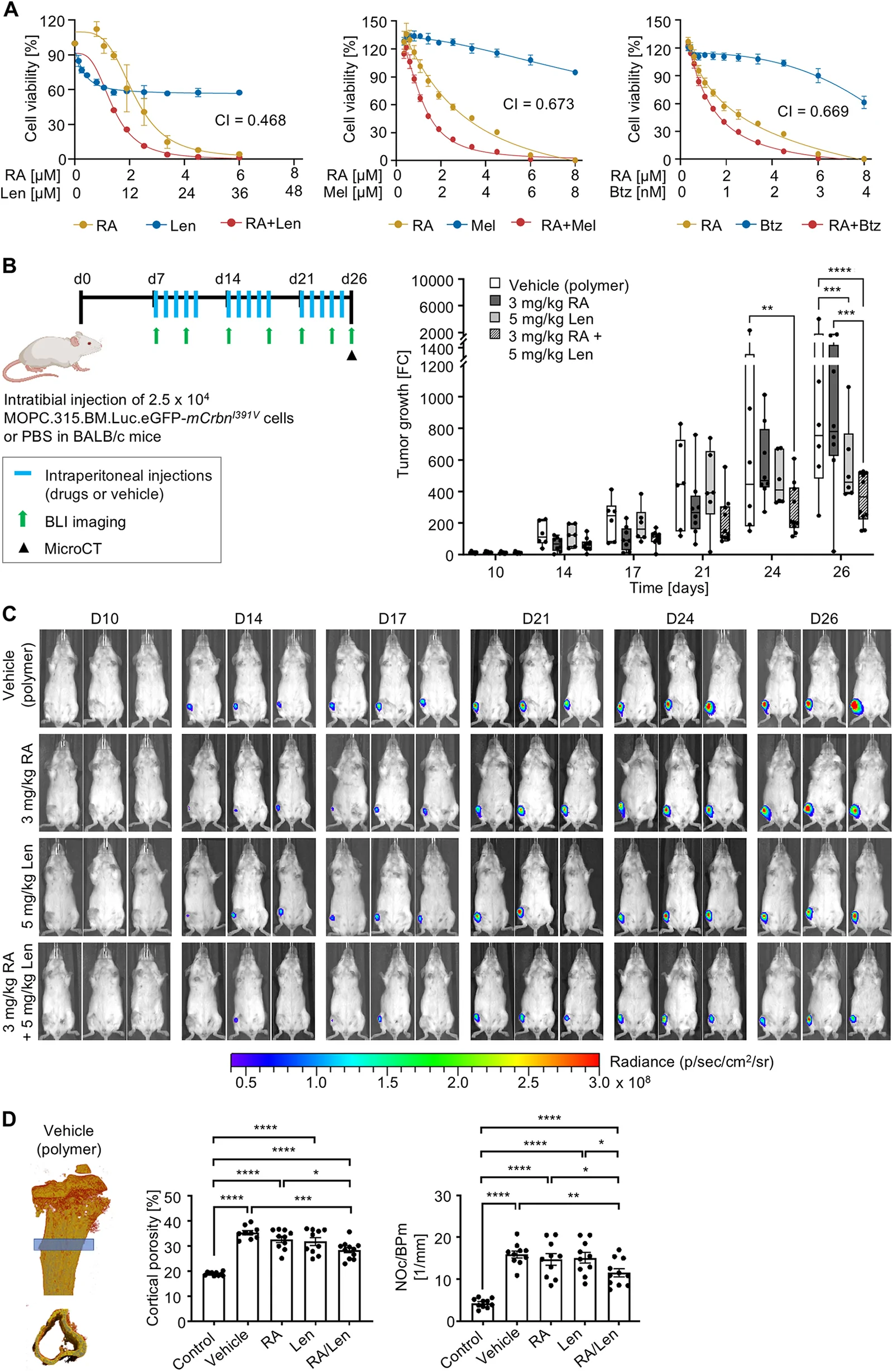
RA controls tumor growth and bone disease. (**A**) Dose-response curves of single drugs alone and corresponding drug combinations with fixed drug concentration ratios (RA: Len = 1:6, RA: Mel = 1:1, RA: Btz = 2:1). The CI value represents the combination index at 50% of inhibition under drug treatment. Data are presented as the mean ± SD of technical triplicates. (**B**) Experimental design of the mouse study. Seven days after intratibial injection of 2.5 × 10^4^ MOPC.315.BM.Luc.eGFP-*mCrbn*^*I391V*^ cells or PBS, mice were treated with 3 mg/kg RA, 5 mg/kg Len, or the combination five times per week for three weeks. BLI imaging (green arrows) and microCT analysis (black triangle) were performed on the days indicated. BLI signals show that RA and Len control tumor growth. Data are shown as fold changes (FC) of total flux at each time point normalized to day 7. Data are presented as the median ± the interquartile range. Whiskers show min to max values. A two-way ANOVA was used for statistical analysis. (C) BLI images of three representative mice at indicated time points after MM injection from ventral view. (**D**) Three-dimensional reconstructions of the metaphyseal region of the tibiae (coronal and transversal view) at day 26 after injection of MOPC.315.BM.Luc.eGFP-*mCrbn*^*I391V*^ cells and treatment with vehicle (polymer). Cortical porosity and number of osteoclasts per bone perimeter (NOc/BPm) in the metaphyseal region of the tibiae at day 26 were determined by microCT analysis and TRAP staining. Values are shown as the mean ± SEM. An ANOVA test with Tukey’s post hoc correlation was used for statistical analysis. *P-value ≤ 0.05, **P-value ≤ 0.01, ***P-value ≤ 0.001, ****P-value ≤ 0.0001

To determine drug ratios of RA, Len, Mel, and Btz that will enhance the efficacy of RA through decreasing the viability of Len-sensitive MOPC.315.BM.Luc.eGFP-*mCrbn*^*I391V*^ cells, we performed viability assays in vitro using drug combinations at different ratios (Fig. 8A). We determined the combination index (CI) for RA-Len (CI = 0.468), RA-Mel (CI = 0.673), and RA-Btz (CI = 0.669), demonstrating enhanced toxicity of all three drug combinations (red lines) compared to RA alone (yellow lines, Fig. 8A).

For administration in vivo, we used the combination of RA and Len at a 1:6 ratio and developed an aqueous nanoformulation based on micelles of a poly(2-oxazoline)s- and poly(2-oxazines)s (POx/POzi) based ABA triblock copolymer i.e. A-pBuOzi-A (where A = hydrophilic, poly(2-methyl-2-oxazoline) and B = hydrophobic poly(2-*n*-butyl-2-oxazine), supplemental Fig. 7–9) to improve the therapeutic efficacy. Remarkably, A-pBuOzi-A enabled the solubilization of up to 10 g/L RA and 3 g/L Len in aqueous media, while maintaining excellent cytocompatibility in vitro (supplemental Fig. 8–9). Cytotoxicity of the drug-loaded polymer micelles was equal to that of DMSO-dissolved drugs in MOPC.315.BM.Luc.eGFP-*mCrbn*^*I391V*^ cells (supplemental Fig. 9), as demonstrated for these drug carriers in other cell lines [35, 47, 48]. 

For in vivo studies, we injected Len-sensitive MOPC.315.BM.Luc.eGFP-*mCrbn*^*I391V*^ cells intratibially into BALB/c mice. We selected the intratibial injection mode to achieve reliable and reproducible engraftment of MM cells directly within the bone marrow microenvironment. This approach minimizes variability associated with MM-cell trafficking and homing. Moreover, establishing a defined tumor burden at a specific skeletal site allowed us to assess subsequent treatment effects on established MM cell tumors and their interaction with the bone microenvironment. After seven days, polymer (vehicle), RA, Len, or the combination was administered five days per week for three weeks (Fig. 8B). Tumor growth was monitored using BLI imaging and revealed that the combination of RA and Len significantly reduced tumor growth from day 24 onwards compared to vehicle-treated and RA-treated mice (Fig. 8B, C). No serious adverse effects such as weight loss were observed. However, pharmacokinetic and biosafety assessments for the RA polymers were not performed. To analyze the potential of RA in controlling osteolytic bone disease, we performed microCT and histological analyses. Three-dimensional reconstructions of the metaphyseal region of the tibiae revealed preserved cortical and trabecular bone structures in control mice, which were intratibially injected with PBS, whereas the trabeculae were destroyed and the tibia plateau was dislocated in MM-injected mice treated with vehicle (polymer; Fig. 8D). Consistently, osteoclast number and activity were significantly increased in vehicle-treated MM mice (Fig. 8D, supplemental Fig. 10). A lesion score was assigned based on number, size, and location of the osteolytic lesions. Most vehicle-treated mice had the highest score [6], whereas a lower score was observed in mice treated with RA and Len (supplemental Fig. 10). Bones of mice treated with RA and Len showed a lower cortical porosity, a higher ratio of bone volume to total volume in a defined region of interest, and fewer osteoclasts compared to vehicle-treated mice (Fig. 8D, supplemental Fig. 10). Our data show that treatment with RA effectively combats tumor growth and osteolytic bone disease in combination with Len.

### RA kills multi-refractory, high-risk patient MM cells

**Fig. 9:**
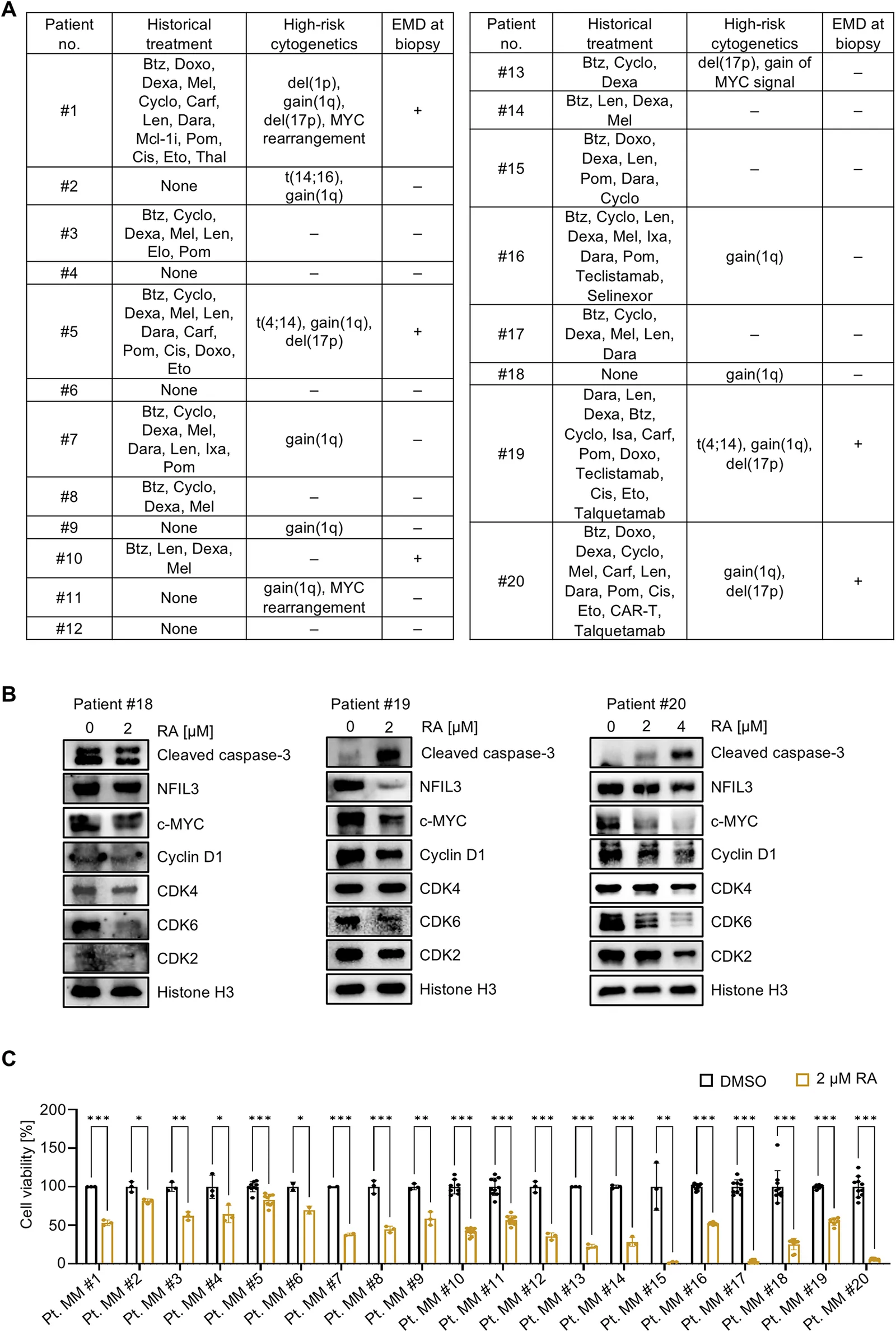
RA blocks the NFIL3-MYC axis and kills multi-refractory high-risk patient MM cells. (**A**) Historical treatment, high-risk cytogenetics, and EMD at biopsy of 20 patients with MM. Btz, bortezomib; Doxo, doxorubicin; Dexa, dexamethasone; Mel, melphalan; Cyclo, cyclophosphamide; Carf, carfilzomib; Len, lenalidomide; Dara, daratumumab; Mcl-1i, Mcl-1 inhibitor (MIK665); Pom, pomalidomide; Cis, cisplatin; Eto, etoposide; Thal, thalidomide; Elo, elotuzumab; Ixa, ixazomib; Teclistamab, Talquetamab, CAR-T, chimeric antigen receptor T cells. (**B**) Immunoblots of whole extracts of patient MM cells (#18, #19, #20) after treatment with RA for 24 h. Blots were probed with antibodies against cleaved caspase-3, caspase-3, NFIL3, c-MYC, Cyclin D1, CDK4, CDK6, CDK2, and Histone H3. (**C**) Cell viability of CD138-positive human primary MM cells isolated from the iliac crest of 20 patients. Cells were treated with DMSO or 2 µM RA for 48 h. Viability was determined using the CellTiter-Glo^®^ Luminescent Cell Viability Assay. Unpaired *t*-tests were used for statistical analysis. Data are presented as the mean ± SD of technical triplicates. *P-value ≤ 0.05, **P-value ≤ 0.01, ***P-value ≤ 0.001, ****P-value ≤ 0.0001

To test the effectiveness of RA in killing patient MM cells, we isolated CD138-positive primary MM cells from the iliac crest of 20 patients. Biopsy samples were collected at diagnosis, at relapse, at refractory disease, and in remission. Details are given in supplemental Table 8. In addition, high risk-cytogenetic abnormalities such as del(17p), TP53, gain(1q), t(4;14), t(14;16) or t(14;20) and MYC rearrangements or gain of the MYC signal were determined in 11/20 patients (Fig. 9A, supplemental Table 8). Prior lines of therapy ranged from no prior line (at diagnosis) up to 13 lines (supplemental Table 8). Heavily pretreated patients were penta- or even multi-refractory with three patients having received BCMA-directed immunotherapies (patient #16, #19, #20). In addition, progressive disease in patients with relapse or refractory disease showed EMD at biopsy (patient #1, #5, #10, #19, #20). RA treatment induced cleaved caspase-3 activity and downregulated the expression of NFIL3, c-MYC, cyclin D1, CDK4, CDK6, and CDK2 in patient sample #19 und #20 as evidenced by immunoblotting, indicating the same regulatory pattern of the NFIL3-MYC axis as in human and murine MM cells (Fig. 9B). Notably, RA reduced the viability of all MM samples regardless of the number of previous lines of therapy (Fig. 9C). In some cases, RA showed an additive effect with Len, Mel, and Btz (supplemental Fig. 11). Furthermore, viability of purified CD34-positive stem cells from autologous stem cell transplants was only slightly decreased, suggesting a therapeutic window for the use of RA (supplemental Fig. 12). These data highlight that RA has potent anti-myeloma activity even in patient-derived high-risk MM cells following failure of BCMA-directed immunotherapies.

## Discussion

**Fig. 10:**
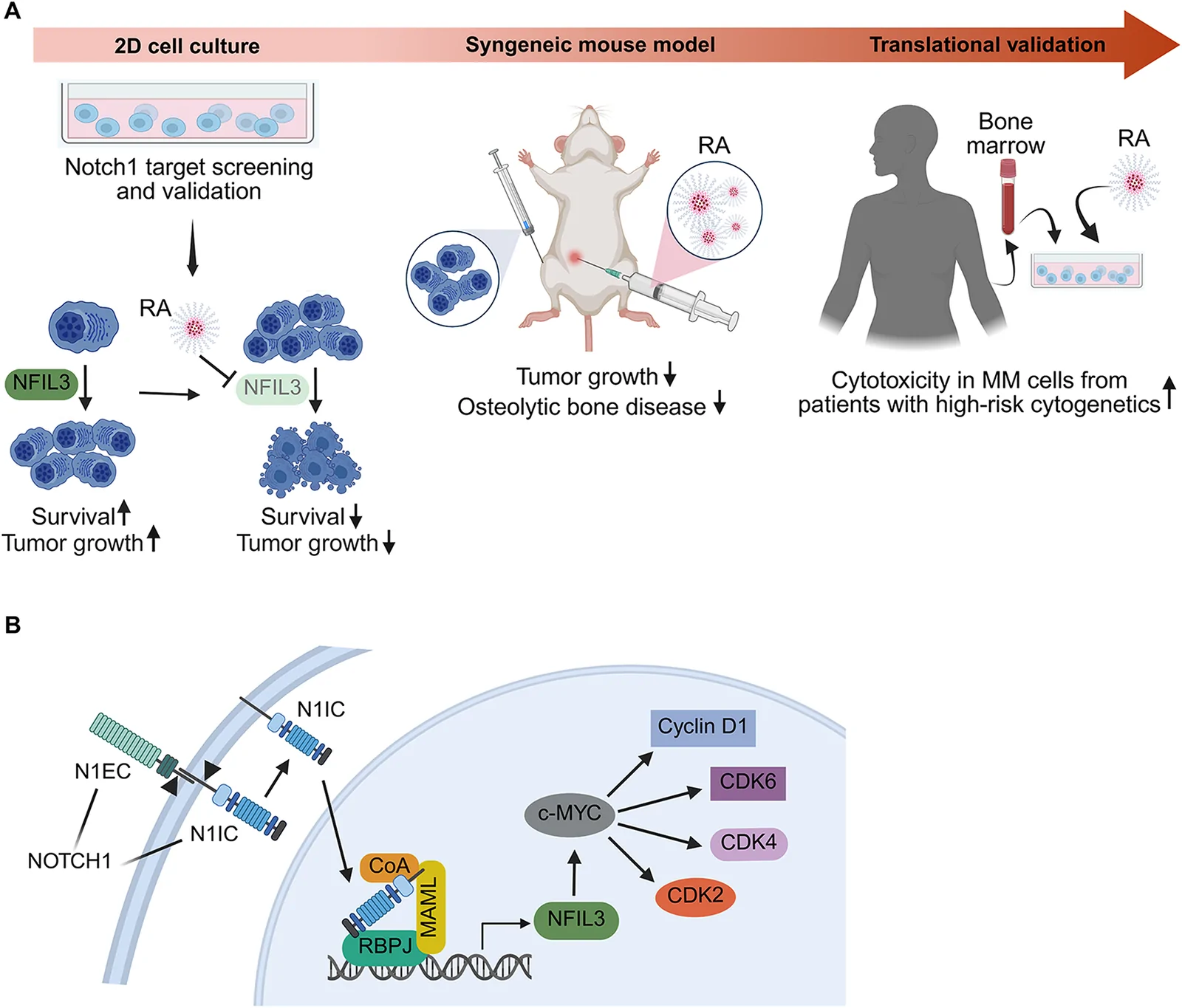
Validation of NFIL3 as a novel therapeutic target and proposed mechanism of the NFIL3-c-MYC axis in MM. (**A**) Schematic overview of the experimental workflow. Notch1 target screening in 2D MM cell culture identified NFIL3 as a regulator of MM cell survival and tumor growth. Pharmacological treatment with RA reduced NFIL3-associated tumor-promoting effects, decreased tumor growth and osteolytic bone disease in a syngeneic mouse model, and induced killing of primary MM cells from patients with high-risk cytogenetics. (**B**) Mechanistic diagram of Notch1 intracellular domain (N1IC) associating with RBPJ, MAML, and coactivators to promote NFIL3 transcription. NFIL3 enhances c-MYC signaling, which in turn supports expression of the cell cycle regulators cyclin D1, CDK6, CDK4, and CDK2, thereby promoting MM cell proliferation and survival

We identified the NFIL3-MYC axis as a novel molecular driver in the high-risk patient cohort with gain/amp(1q) and demonstrated that RA targeting this pathway overcomes resistance in multi-refractory MM cells with high-risk cytogenetics (Fig. 10). Additionally, we developed RA nanocarriers that suppressed MM cell growth and diminished osteolytic bone disease in the MOPC315.BM model (Fig. 10).

In an unbiased approach, we performed RNAseq of Notch-depleted MM cells. GSEA revealed that IRF4 and MYC were among the most downregulated pathways. The two key transcription factors activate each other through an autoregulatory loop, which is indispensable for MM growth and survival [42, 49]. We selected 40 downstream Notch effectors based on their druggability by small molecule inhibitors or targeted immunotherapeutics. We used high-throughput functional genomic screens with shRNA libraries to identify those candidates that mediate multidrug resistance against Len, Mel, and Btz in MM cells with high Notch activity (Fig. 10). Bioinformatics analysis identified the basic leucine zipper transcriptional repressor E4 promoter-binding protein (E4BP4) or NFIL3 [50], as the most consistent hit of the screens, conferring resistance towards all three drugs.

NFIL3 activates the interleukin-3 (IL-3) promoter in human T cells [51]. Using the publicly available data set of the MMRF CoMMpass study cohort, we showed that high NFIL3 levels confer inferior survival and are associated with the high-risk cytogenetic abnormality gain/amp(1q). In a multivariate survival analysis, high NFIL3 levels were verified as an independent prognostic marker for shorter patient survival regardless of their association with high-risk cytogenetics in this study. In the MMRF cohort and in an independent validation cohort, we found that NFIL3 is upregulated in patients with a higher TMC. In the validation cohort, higher NFIL3 levels are associated with a higher incidence of EMD at biopsy, a clinical condition that is associated with a highly aggressive disease course, indicating that NFIL3 is an important factor in MM progression, relapse, and therapy resistance. Similarly, higher expression of NFIL3 has been linked to an inferior prognosis in triple negative breast cancer or in colorectal cancer, promoting progression and metastasis [50]. 

Next, we provided evidence that expression of NFIL3 in MM is transcriptionally induced by activated Notch1 (N1ΔE) in U266 cells. In addition, ChIP-seq analysis with antibodies against RBPJ, a transcriptional co-activator of the intracellular domain of the Notch1 receptor after proteolytic cleavage at the membrane, revealed binding to the canonical Notch target gene *HEY1* and to the promoter of *NFIL3*. Reporter assays with U266-N1ΔE cells overexpressing Notch1 confirmed that NFIL3 is a downstream target of canonical Notch1/RPBJ signaling in MM cells.

To study downstream effects of NFIL3 on oncogenic pathways and MM cell viability and survival, we generated NFIL3-depleted MM.1S cells and used the drug RA, a natural bioactive compound isolated from Anemone raddeana Regel [52]. RA is an oleanane-class triterpenoid saponin that suppresses growth and induces apoptosis of colorectal cancer cells, by inhibiting the Wnt/β-catenin pathway with the downstream target c-MYC and cyclin D1 [53]. Recently, RA has been shown to have anti-tumor effects in MM cell lines, inducing G2 cell cycle arrest and mitochondrial-pathway-dependent apoptosis via the MAPK/ERK signaling pathway [54]. In our study, depletion of NFIL3 in MM cells inhibited the MYC transcriptional program including c-MYC, cyclin D1, CDK4, CDK6, and CDK2. In parallel, viability and proliferation of MM.1S cells were reduced after NFIL3 depletion alone and in combination with Len, Mel, and Btz, indicating that NFIL3 mediates drug resistance in MM cells. Similarly, RA blocked the MYC transcriptional program and induced apoptosis in MM.1S cells as evidenced by increased cleaved caspase-3 expression. RA alone and in combination with Len, Mel, and Btz reduced cell viability and growth of MM cells. Interestingly, higher levels of NFIL3 and c-MYC in patient samples of the validation cohort correlated with a higher mutational count, which has been associated with tumor progression and poorer survival in different cancer types [55]. 

NFIL3 may promote resistance to mechanistically distinct anti-myeloma agents by regulating a common cellular survival program rather than interfering with their individual drug targets. NFIL3 has previously been characterized as a survival factor that restricts FOXO-dependent transcription of pro-apoptotic and stress-response genes, including TRAIL, FAS and GADD45 family members, thereby protecting tumor cells from stress-induced apoptosis [56]. Although Len, Mel and Btz exert their primary effects through CRBN-dependent protein degradation, DNA damage and proteasome inhibition, respectively, these pathways ultimately converge on the induction of cellular stress and apoptosis [57–59]. Our observation that NFIL3 depletion reduces MYC expression and enhances drug-induced cell death therefore suggests that NFIL3 increases the apoptotic threshold and provides a shared survival advantage across different therapeutic classes. Consistent with this concept, maintenance of MYC-dependent transcription has been identified as an important mechanism of therapeutic resistance in MM [60]. Thus, our data support NFIL3 as a potential common modulator of drug sensitivity rather than implying three independent drug-specific resistance mechanisms.

Similar results were obtained in murine Len-sensitive MOPC315.BM cells (MOPC.315.BM.Luc.eGFP-*mCrbn*^*I391V*^*)*, indicating that RA collapses a MYC-driven transcriptional program in human and murine MM cells (Fig. 10). Determination of the combination index of RA and Len in vitro with murine MM cells revealed a synergistic toxicity of the drug combination that was confirmed by our in vivo results. RA alone or in combination with Len controlled MM cell growth and had a bone-protective effect, blocking osteoclast development and activity in the MOPC.315.BM.Luc.eGFP-*mCrbn*^*I391V*^ mouse model. Our results confirmed the effect of RA on osteolytic lesions in a breast cancer model, in which RA inhibited growth, and invasion of breast cancer cells, and the development of osteolytic lesions [61]. To improve therapeutic efficacy, we developed a nanoformulation of RA and Len based on POx/POzi triblock copolymers. Because both drugs are highly hydrophobic and exhibit poor aqueous solubility, we formulated them into aqueous micelles using a triblock copolymer (A-pBuOzi-A). Using the same or similar amphiphiles, nanoformulations with exceptional properties including ultra-high drug loading and excellent cyto- and biocompatibility have been generated [35, 47, 48, 62], and have been excellently tolerated following in vivo administration, either intravenously, intraperitoneally, or subcutaneously [63, 64]. We used the triblock copolymer A-pBuOzi-A as it allowed for high drug loading for Len and RA, exhibited optimal cytocompatibility, and had no apparent toxicity in mice. Although our data show promising results, further optimization will be required to support the translation of these nanocarriers into clinical development. Additional studies are needed to determine whether the nanocarriers can overcome physiological barriers in humans to achieve sufficient tumor accumulation and efficient cellular internalization. However, formulations similar to those introduced here have been shown to successfully treat medulloblastoma and can undergo endocytosis very rapidly [65, 66]. In addition, high-dose paclitaxel formulations have been reported to trigger immunogenic cell death [66]. Interestingly, RA elicited tumor immunogenicity and enhanced antitumor immunity by dendritic cell-mediated antigen cross-presentation and T cell activation in melanoma and colon adenocarcinoma models [67]. In combination with immune checkpoint therapies, RA enhanced the efficacy of cancer immunotherapy, indicating its rationale use as a promising anticancer immunotherapeutic treatment [67]. 

Gain/amp(1q) is a high-risk cytogenetic event that is highly correlated with a higher proliferation index and with a higher risk of relapse in MM [68]. Our results highlight that RA is effective in patient samples with high-risk cytogenetic events. Even in penta-refractory patients after BCMA-directed immunotherapies including CAR-T cells and bispecific antibodies, RA effectively killed MM cells and collapsed the NFIL3-MYC driven transcriptional program. Following these lines of evidence, RA might overcome drug resistance in MM, particularly at sides of T cell exhaustion as in extramedullary disease [69] to improve low clinical responses after immunotherapies.

This study presents valuable findings, however several aspects merit further consideration. At the mechanistic level, NFIL3 depletion consistently suppressed the MYC-associated transcriptional program. It would be worthwhile to explore direct transcriptional regulation of MYC by NFIL3 through complementary approaches such as promoter occupancy and rescue experiments. Additionally, although RA showed substantial anti-myeloma activity, its pharmacological specificity for NFIL3 remains to be fully established, and potential NFIL3-independent effects cannot be excluded. Moreover, the in vivo efficacy of RA was evaluated in a single murine MM model, as well-validated Len-sensitive syngeneic MM models remain limited and have only become available recently. In addition, comprehensive pharmacokinetic, biodistribution, and long-term toxicity studies of the RA nanoformulation would need to be performed before considering it for use in translational studies focusing on drug development. Lastly, the analyses of primary MM cells were performed in a relatively small and clinically heterogeneous patient cohort, including only a limited number of patients previously exposed to BCMA-directed immunotherapies, which warrants validation in larger independent cohorts. Collectively, these limitations highlight the need for additional mechanistic and translational studies before therapeutic targeting of the NFIL3–MYC axis can be advanced toward clinical application.

## Conclusions

We identified NFIL3 as a novel druggable Notch effector and developed biopolymer-based RA nanocarriers targeting the NFIL3-MYC axis in multidrug-resistant MM cells. We provide an innovative pharmaceutical strategy inhibiting Notch downstream signaling in multi-refractory MM with high-risk cytogenetic abnormalities to overcome drug resistance and improve patient outcome in clinical trials.

## Supplementary Information

Below is the link to the electronic supplementary material.

Supplementary Material 1

Supplementary Material 2

Supplementary Material 3

## Data Availability

The datasets (RNAseq and ChiP-seq) supporting the conclusions of this article are availableat GEO (accession number GSE215091, https://www.ncbi.nlm.nih.gov/geo/query/acc.cgi? acc=GSE215091) and at the European Genome-Phenome Archive (accession number EGAS50000000392, https://ega-archive.org/studies/EGAS50000000392). For other original data, please contact jundt_f@ukw.de. Additional details on methods and data supporting the key findings are included within the supplemental data files.

## Abbreviations

3Rs: Replacement, reduction, refinement
BCMA: B-cell maturation antigen
BM: Bone marrow
Btz: Bortezomib
CAR-T: Chimeric antigen receptor T cell
CDK: Cyclin-dependent kinase
ChIP-seq: Chromatin immunoprecipitation sequencing
CI: Combination index
CIs: Confidence intervals
CTGF: Connective tissue growth factor
CXCL12: C-X-C motif chemokine ligand 12
DMSO: Dimethyl sulfoxide
DNA: Deoxyribonucleic acid
E4BP4: E4 promoter-binding protein
EDTA: Ethylenediaminetetraacetic acid
EMD: Extramedullary disease
FC: Fold change
GAPDH: Glyceraldehyde 3-phosphate dehydrogenase
GSEA: Gene set enrichment analyses
HES1: Hairy and enhancer of split-1
HES6: Hairy and enhancer of split-6
HEY1: Hes-related family bHLH transcription factor with YRPW motif 1
HPRT: Hypoxanthine-guanine phosphoribosytransferase
HRs: Hazard ratios
i.p.: intraperitoneal
i.t.: intratibial
IL-3: Interleukin-3
IRF4: Interferon regulatory factor 4
Len: Lenalidomide
MAF: Musculoaponeurotic fibrosarcoma
MAPK/ERK: Mitogen-activated protein kinase/Extracellular signal-regulated kinase
mCrbn: murine Cereblon
Mel: Melphalan
microCT: micro-computed tomography
MM: Multiple myeloma
MMRF: Multiple Myeloma Research Foundation
mRNA: Messenger RNA
NES: Normalized enrichment scores
NFIL3: Nuclear Factor, Interleukin 3 Regulated
NGS: Next-generation sequencing
Notch: Neurogenic locus notch homolog protein
PBS: Phosphate-buffered saline
PFA: Paraformaldehyde
PID: Pathway interaction database
pStat3: Phospho-Stat3
qPCR: Quantitative PCR
RA: Raddeanin A
RBPJ: Recombination signal-binding protein for immunoglobulin kappa J region
RIPA buffer: Radioimmunoprecipitation assay buffer
RNA: Ribonucleic acid
RNAseq: RNA sequencing
ROI: Region of interest
SDS: Sodium dodecyl sulfate
shRNA: Short hairpin RNA
Stat3: Signal transducer and activator of transcription 3
TMC: Tumor mutational count
TPM: Transcripts per million
TRAP: Tartrate-resistant acid phosphatase
WNT5B: Wnt family member 5B
