# Supplementary material for: The NFIL3-MYC signaling axis: a novel therapeutic target and prognostic marker in high-risk multiple myeloma
Source: Biomark Res. 2026 Oct 9;14:135. doi: 10.1186/s40364-026-01018-7 (PMC13651131; doi:10.1186/s40364-026-01018-7)
Supplement: Supplementary file 1 [file 40364_2026_1018_MOESM2_ESM.docx]

**Supplemental data**

**The NFIL3-MYC signaling axis: A novel therapeutic target and prognostic marker in high-risk multiple myeloma**

Daniela Simone Maichl, Wen-Hui Cheng, Poonam Kumari, Julius Arthur Kirner, Stefanie Schmidt, Carsten Patrick Ade, Mengshi Yang, Malik Salman Haider, Yuquan Chi, Robert Luxenhofer, Lyudmyla Taranets, Melanie Haffner-Luntzer, Verena Fischer, Anita Ignatius, Ellen Leich, Gerardo Zapata, Nikita Popov, and Franziska Jundt

**Supplemental Methods**

**Primary samples**

Stem cell samples were obtained from patients with multiple myeloma (MM) undergoing autologous stem cell collection (after stem cell mobilization chemotherapy and under stimulation with filgrastim). Stem cell collection was performed with the Spectra Optia apheresis system (Terumo BCT, Lakewood, CO, USA; software version 11.3) and the according IDL set. The inlet ratio of anticoagulant solution ACD-A to whole blood was 1:12. The samples were drawn during the procedure from the collection bag under sterile conditions using the attached additional sample bag for quality controls. The separated cells were resuspended in RPMI 1640 medium containing 10 % FBS, counted, and seeded at a density of 2 × 10^5^ cells/ml in a 96 well plate (100 µl/well). Samples were immediately treated in triplicates for 48 h. Cell viability was analyzed using the CellTiter-Glo® Luminescent Cell Viability Assay (Promega, Fitchburg, WI, USA) and a Luminoskan Ascent Microplate Luminometer (Thermo Fisher Scientific, Darmstadt, Germany) according to the manufacturer’s protocol.

**Generation of U266 cell clones with constitutively active NOTCH1**

U266 cells with low amounts of Notch1 (N1) were used to introduce expression of the Notch1 variant N1ΔE that represent a constitutively active Notch1 form.^1^ N1ΔE contains the intracellular domain of the receptor and the transmembrane domain that leads to ligand-independent, but γ-secretase-dependent Notch activation, but lacks the extracellular domain.^1^ To this end, RNA was extracted from MM.1S cells and transcribed into cDNA as previously described. The cDNA served as a template for amplification of the intracellular NOTCH1 domain (N1IC) in a standard PCR reaction (Phusion Hot-Start II High-Fidelity DNA Polymerase, Thermo Fisher Scientific) according to the manufacturer's instructions. The NΔE fragment was then synthesized as two single fragments. The short transmembrane sequence (N1ΔE-SS) was ordered from Sigma-Aldrich as single stranded oligonucleotide and the reverse strand was added via a standard PCR reaction. The long fragment (N1ΔE-LS) was amplified using MM.1S cDNA as template and followed by restriction digest and ligation of the two fragments to the final NΔE sequence. XbaI restriction sites were introduced by PCR during amplification of all fragments and used for cloning of the NOTCH1 fragments into the lentiviral expression vector pCDH-EF1α-MCS-IRES-Neo according to the manufacturer’s protocol. Plasmid vectors used in this study are listed in supplemental Table 1. U266 wells were transduced with the vector by spinoculation and selected by adding 1,000 µg/ml G418 to the cell culture medium.

**Flow cytometry**

For analysis of N1ΔE expression, cells were intracellularly stained. Therefore, cells were washed with washing solution (PBS containing 5 % FCS), fixed with 4 % PFA in PBS for 10 min and permeabilized with 90 % methanol on ice for 30 min. Cells were then incubated with rabbit anti-Notch1 (Cell Signaling Technology, Frankfurt am Main, Germany) primary antibodies for 1 h at room temperature (RT), washed and incubated with the secondary antibody (1:500, Alexa Fluor 647 coupled anti-rabbit, Thermo Fisher Scientific) for 1 h at RT. After another washing step, cells were resuspended in PBS and flow cytometric data was acquired on a FACSCanto™ II (BD Biosciences, Heidelberg, Germany). Data was analyzed using the software Flowing v2.5.1 (Cell Imaging Core, Turku Center for Biotechnology, Turku, Finland). Purified CD138‑positive primary MM cells were stained with the FITC-conjugated anti-CD138 antibody (Biolegend, San Diego, CA, USA) according to the manufacturer’s instructions.

**Bioinformatic analysis and selection of Notch1 target genes for the tailored shRNA library**

Processing of RNAseq raw data was performed using a custom pipeline that included edgeR for calling the differential expression. RNAseq data are available at GEO under accession number GSE215091.

13,730 genes were evaluated by their strength and consistency of regulation. Therefore, an adjusted *P*‑value cutoff (*padj* < 0.05) was applied in the condition GSI XII versus DMSO. The remaining genes were scored according to their direction of regulation (log_2_FC) for each condition: GSI XII versus DMSO, shN1 #1 versus empty vector (EV), shN1 #2 versus EV. Because genes regulated in the shN1 #2 and GSI XII treatment conditions showed a higher correlation, we compared all significantly downregulated genes across these two conditions, and further evaluated the overlapping genes based on literature search focusing on relevance to MM pathogenesis and druggability. 40 downregulated *NOTCH1* target genes were selected for the generation of a focused shRNA library screen (supplemental Table 2). Venn diagrams for significantly (*padj* < 0.05) *NOTCH1* up- and downregulated genes were created using Venny 2.1.0.^2^ For overlapping up- and downregulated genes, a heatmap was created using the online tool Morpheus (https://software.broadinstitute.org/morpheus).

**Generation of a pooled shRNA library for the functional genomics screens**

A focused shRNA library was used to decrease experimental noise and increase the chance of identifying statistically significant hits. The shRNA library contained a small set of equally represented and highly efficient shRNAs specifically targeting the 40 previously selected Notch1 target genes (supplemental Table 2). Library generation was performed as previously described.^3^ For each of the 40 genes, five individual miR-E shRNAs were selected according to Sensor-based^4^ and SplashRNA-based^5^ predictions and commercially synthesized (Integrated DNA Technologies (IDT), Coralville, IA, USA) as single stranded 97-mer oligonucleotides in a 386-well plate format (supplemental Table 3). The oligos were pooled equimolarly and amplified in batch using the Phusion™ High-Fidelity DNA polymerase (Thermo Fisher Scientific) and PCR primers that introduce EcoRI and XhoI restriction sites.^3^ The amplified sequences were cloned in batch into the doxycycline-inducible target vector pLT3GGmirEPPIR^4^ using a standard T4 DNA ligase (New England Biolabs, Frankfurt, Germany) reaction. Following transformation of XL1-Blue *E. coli* cells and amplification on LB agar plates, more than 2 × 10^5^ individual bacterial colonies were harvested, combined, and directly used to prepare the plasmid DNA using the NucleoBond Xtra Maxi kit (Macherey-Nagel) according to the manufacturer's protocol. This procedure guaranteed a largely equal distribution of all shRNAs in the final plasmid pool, which was verified by next-generation sequencing (NGS) using an Illumina NextSeq 500 sequencing system (supplemental Figure 2). In the final step, the plasmid pool was used to produce a pooled lentiviral shRNA library, and the engineered U266 cell line U266-N1ΔE was transduced by spinoculation. Infection was performed at a moity of infection of 0.1, which guaranteed single-copy integrations of the individual shRNAs. To verify knockdown efficiency of the shRNAs in the library, the reduction of target protein was validated for several shRNAs by immunoblot analysis. ShRNAs for the known *NOTCH* target genes *HEY1* and *HES6*, eight individual shRNAs targeting luciferase as negative control and shRNAs targeting ubiquitin B and polo like kinase 1 as controls for cell death were included in the shRNA library.

**Functional genomics screens and selection of screen hits**

Functional genomics screens were carried out as negative selection screens using three biological replicates of the shRNA library-transduced U266-N1ΔE cell line. Prior to the start of the screens, the cells were pre-treated with doxycycline (1 µg/ml) for 24 h to induce shRNA expression. Cells were then treated with DMSO or with Len (1 µM), Mel (1.25 µM), or Btz (0.8 nM) as selective pressure. At day 0 and 12, the cells were harvested and the genomic DNA was extracted using the NucleoSpin Tissue Mini kit (Macherey-Nagel). ShRNA sequences were recovered from the genomic DNA using a two-step custom PCR approach to introduce binding sites for hybridization to the Illumina flow cell and to introduce indices for multiplexing as described.^3^ The final 257 bp long PCR product was purified using the NucleoSpin Gel and PCR Clean‑up Mini kit (Macherey-Nagel). The concentration and quality of the samples were analyzed using the 5200 Fragment Analyzer System (Agilent). Changes in shRNA frequencies were detected by NGS using an NextSeq 500 System (Illumina) and bioinformatic analyses were performed as previously described.^6^ In short, BCL files were transformed into FASTQ files using the bcl2fastq Conversion Software v1.8.4 (Illumina). The software Bowtie v0.12.8 was used to align the guide-stem sequences to a reference dataset containing the guide-stem sequences of all library-specific shRNAs.^7^ Raw counts per shRNA were normalized by calculating counts per million (CPM) values. ShRNA sequences with an average baseMean (DESeq) below 160 were excluded from further analysis. For calculation of changes of frequencies of each shRNA, pools were analyzed in triplicates at day 12 with cells expressing the shRNA library and were either treated with Len, Mel, Btz, or with DMSO. Depletion and enrichment of shRNAs was represented as log_2_FC and for each target gene the median of the log_2_FCs of all shRNAs was determined. Z-scores were calculated based on the median values by subtracting the average of all medians and dividing the result by the standard deviation of all medians (supplemental Table 7). A z-score cutoff of −0.5 was applied and target genes were scored regarding their number of hits among all four screens. The sequencing data for the functional genomics screens are available at GEO under the accession number GSE215091.

**MOPC.315.BM.Luc.eGFP-*mCrbn^I391V^* mouse model**

The well-established MOPC.315.BM MM mouse model was used in combination with recently generated Len-sensitive MOPC.315.BM.Luc.eGFP-*mCrbn^I391V^* cells.^8^ These cells were generated through retroviral expression of a mutant murine cereblon in MOPC.315.BM cells, after replacing the isoleucine at position 391 with valin (mCrbnI391V).^8^ MOPC.315.BM.Luc.eGFP-*mCrbn^I391V^* cells are Len-sensitive since the mutant form Crbn^I319V^ of cereblon enables Len-induced degradation of Ikaros transcription factors and decreases cell viability.^8^ Seven-week old female BALB/c mice (BALB/cAnCrl) were obtained from Charles River Laboratories (Sulzfeld, Germany) and acclimatized for 10 days in the animal facility of the University Hospital Würzburg. For intratibial (i.t.) injections of MM cells, mice were anaesthetized via intraperitoneal (i.p.) injection of 100 mg/kg ketamine and 10 mg/kg xylazine in PBS. Subsequently PBS (control) or 2.5 × 10^4^ MOPC.315.BM.Luc.eGFP‑*mCrbn^I391V^* cells in 10 µl PBS were injected into the medullary cavity of the right tibia. To confirm tumor engraftment and monitor tumor progression, bioluminescent imaging (BLI) was performed at days 3, 7, 10, 14, 17, 21, 24, and 26. Therefore, mice were anaesthetized with isoflurane (3-4 % for induction, 2-3 % for maintenance) after i.p. injection of luciferin (300 mg/kg). BLI images were acquired 10 min after luciferin injection on an IVIS Spectrum imaging system (PerkinElmer, Massachusetts, USA) and analyzed using the Living Image 4.7 software (PerkinElmer).

**Preparation and quantification of drug-loaded micellar formulations**

The amphiphilic biocompatible triblock copolymer PMeOx_35_-*b*-PBuOzi_20_-*b*-PMeOx_35_, where MeOx is poly(2-methyl-2-oxazoline) and BuOzi is poly(2-n-butyl-2-oxazine), also represented as A-pBuOzi-A, was used to develop the micellar formulations. The details of polymers synthesis and its characterization were previously published.^9^ The thin film hydration method was used to prepare the drug-loaded polymer micelles.^10^ Specifically, methanolic stock solutions of the drugs (5 g/l) and the polymer (10 g/l) were mixed in the indicated ratios, followed by removal of the solvent at 50 °C. Subsequently, the films were dried in vacuo (≤ 0.2 mbar) for 20 min. Pre-warmed (37 °C) H_2_O was added for solubilization while shaking the samples in a Thermomixer comfort (Eppendorf AG, Hamburg, Germany) at 1,250 rpm at 50 °C for 15 min. Samples were centrifuged for 5 min at 7,788 g with a MIKRO 185 (Hettich, Tuttlingen, Germany) to remove non-solubilized drugs. For quantification of the micellar formulations, HPLC analysis was performed and measured values were compared to standard curves (supplemental Figure 7B, D). HPLC samples were prepared by diluting 10 µl of the samples with an appropriate volume of acetonitrile (ACN) and H_2_O mixture (60/40, v/v). For each HPLC measurement, three samples were prepared individually. Analysis was carried out on a LC-20A Prominence HPLC (Shimadzu, Duisburg, Germany), equipped with a system controller CBM-20A, a solvent delivery unit LC-20 AT (double plunger), an online degassing unit DGU-20A, an auto-sampler SIL-20 AC, a photo-diode array detector SPD-M20A, a column oven CTO-20 AC, and a refractive index detector RID-20A. As stationary phase, a ZORBAX Eclipse Plus (Agilent) C18 column (4.6 × 100 mm; 3.5 µm) was used. Samples injection volume was 20 µl. Elution was performed using a mobile phase of H_2_O and ACN containing 0.05 % trifluoroacetic acid (TFA) at 40 °C and a flow rate of 1 ml/min. The proportion of ACN was increased from 40 % to 60 % within the first 10 min for RA. Afterwards, it was increased to 80 % ACN in 0.1 min and kept constant for 1.9 min, followed by a final decrease to the initial proportion of 40 % ACN in 0.1 min (supplemental Figure 7C). The proportion of ACN was kept at 1 % within the first 5 min for Len. Subsequently, it was increased to 99 % ACN in 0.1 min and kept constant for 6.9 min. Finally, it was decreased to the initial proportion of 1 % ACN in 0.1 min. Detection of RA was performed at 203 nm with a retention time of 6.05 min. Len was quantified at 311 nm with a retention time of 7.52 min (supplemental Figure 7C).

To calculate the loading capacity (LC) and loading efficiency (LE) of the formulations, the following equations were used:

$LC=\frac{m_{drug}}{m_{drug}+m_{excipient}}\times100 \%$	(equation 1)

$LE=\frac{m_{drug}}{m_{drug, added}}\times100 \%$	(equation 2)

where m_drug_ and m_excipient_ are the weight amounts of the solubilized drug and polymer excipient in solution and m_drug,added_ is the weight amount of the drug initially added to the dispersion. No loss of polymer during micelles preparation was assumed. For long-term stability studies, the polymer-drug micelles were prepared (day 0) as described and stored at ambient conditions. Three individual samples were analyzed by HPLC on day 0, 1, 5, and 15 after repeated centrifugation for 5 min at 7,788 g to remove precipitate, if any. In the meantime, samples were stored over their precipitate, if any.

Cytocompatibility of the plain polymer (A-pBuOzi-A) was tested on MM cells (MM.1S, RPMI 8226, AMO-1, MOPC315.BM-*mCrbn^I391V^*). Therefore, cells were treated for 48 h with increasing concentrations of the polymer (up to 5 g/l), dissolved in PBS, and diluted in cell culture medium.

For the in vivo experiments, the micellar formulations were reconstituted freshly from lyophilizate each week and quantified by HPLC after redispersion. The appropriate volume of aqueous micellar formulation was prepared in bulk, aliquoted for the daily usage and lyophilized overnight. After redispersion of the samples with double-distilled H_2_O and dilution with PBS, samples were sterilized by filtering through 0.2 µm syringe filters (Minisart® RC, Sartorius) prior to quantification by HPLC or i.p. injection into the mice. For the polymer-formulated RA, the filters were rinsed with 100 % ethanol and water prior to sample filtration to increase the hydrophilicity of the filters and therefore decrease drug loss.

**Microcomputed tomography (microCT) analysis**

The fixed bones were scanned at RT using a microCT scanning device (Skyscan 1172 v1.5; Skyscan, Kontich, Belgium) at an isotrophic resolution of 8 μm using a peak voltage of 50 kV and 200 μA. Analysis and calibration steps were performed according to the guidelines of the American Society for Bone and Mineral Research.^11^ Two phantoms with a defined density of hydroxyapatite were scanned within each scan for calibration of bone mineral density. Threshold for mineralized tissue was set at 642 mg hydroxyapatite per cm^3^. Analysis was performed with the Skyscan software NRecon, CTAn and CTVox. The region of interest (ROI) for structural analysis was chosen based on integrity of the bone samples, because some were heavily destroyed by the cancer cells. ROI was set in an area where all bone samples could be analyzed. ROI started at 6,000 µm proximal to the distal contact point of the fibula at the tibia, ROI width was 320 µm in the proximal direction. Three-dimensional reconstructions were generated by CTVox and tibia lesion score was assessed according to the following evaluation of the whole bones: 0 – no lesions; 1 – minimal lesions; 2 – more than 3 small lesions; 3 – more than 3 small and additional larger lesions; 4 – more than three large lesions, most lesions located proximal; 5 – more than five large lesions proximal and distal; 6 – more than five large lesions proximal and distal plus a destroyed tibia plateau.

**Supplemental Table 1. Antibiotics used in this study.**

| **Antibiotics** | **Sources** | **Catalog #** | **Applications** |
| --- | --- | --- | --- |
| Puromycin | Thermo Fisher Scientific | A1113803 | Selection of cells transduced with pLKO.1-Puro, pLT3GGmirEPPIR, or pGIPZ |
| G418 | PanReac AppliChem | A6798 | Selection of cells transduced with pCDH-EF1α-MCS-IRES-Neo |

**Supplemental Table 2. RNAseq profile of 40 downregulated genes in MM.1S cells treated with GSX XII and after *NOTCH1* knockdown with shN1 #1 and shN1 #2.**

| **HGNC symbol** | **Gene** | **Entrez Gene ID** | **Log_2_FC GSI XII** | ***padj*** | **Log_2_FC shN1 #1** | ***padj*** | **Log_2_FC shN1 #2** | ***padj*** |
| --- | --- | --- | --- | --- | --- | --- | --- | --- |
| *AFAP1L2* | Actin filament associated protein 1 like 2 | 84632 | -0.3 | 7.3E-01 | -1.2 | 1.8E-02 | -1.2 | 8.5E-03 |
| *ATP2A3* | ATPase sarcoplasmic/endoplasmic reticulum Ca2+ transporting 3 | 489 | -0.5 | 1.7E-03 | 0.0 | 8.4E-01 | -0.5 | 1.3E-03 |
| *CAD* | Carbamoyl-phosphate synthetase 2, aspartate transcarbamylase, and dihydroorotase | 790 | -0.5 | 4.5E-04 | -0.3 | 5.5E-03 | -0.5 | 5.5E-04 |
| *CBFB* | Core-binding factor beta subunit | 865 | -0.3 | 3.7E-02 | 0.0 | 9.4E-01 | -0.5 | 1.6E-03 |
| *CCND1* | Cyclin D1 | 595 | -1.0 | 2.5E-04 | -0.3 | 2.1E-01 | -0.7 | 3.5E-03 |
| *CHST12* | Carbohydrate (chondroitin 4) sulfotransferase 12 | 55501 | -0.8 | 1.1E-06 | 0.0 | 9.4E-01 | -0.7 | 2.3E-06 |
| *CLPTM1L* | Cleft lip and palate transmembrane protein 1-like | 81037 | -0.6 | 2.7E-05 | -0.1 | 6.4E-01 | -0.9 | 1.2E-12 |
| *CTGF* | Connective tissue growth factor | 1490 | -2.4 | 4.1E-03 | -1.9 | 2.3E-02 | -0.4 | 6.8E-01 |
| *CXCR4* | C-X-C motif chemokine receptor 4 | 7852 | -1.0 | 3.5E-04 | -0.4 | 6.6E-03 | -1.0 | 6.9E-10 |
| *DCLRE1C* | DNA cross link repair | 64421 | -0.4 | 1.3E-02 | -0.1 | 8.1E-01 | -0.4 | 1.2E-02 |
| *DOCK10* | Dedicator of cytokinesis 10 | 55619 | -0.7 | 1.1E-06 | -0.4 | 2.9E-03 | -0.4 | 1.6E-02 |
| *DUSP6* | Dual specificity phosphatase 6 | 1848 | -0.7 | 7.8E-04 | -0.3 | 1.6E-01 | -0.8 | 2.1E-04 |
| *ELL2* | Elongation factor for RNA polymerase II 2 | 22936 | -0.4 | 1.2E-02 | -1.1 | 2.0E-18 | -0.2 | 4.0E-01 |
| *ETS1* | ETS proto-oncogene 1, transcription factor | 2113 | -0.7 | 1.3E-06 | -0.4 | 2.6E-03 | -0.6 | 1.5E-04 |
| *ETV4* | ETS variant 4 | 2118 | -0.2 | 8.1E-01 | -1.4 | 5.6E-02 | -1.6 | 2.4E-02 |
| *HES1* | Hes family basic helix loop helix (bHLH) transcription factor 1 | 3280 | -0.6 | 6.3E-02 | 0.2 | 7.7E-01 | 0.2 | 6.2E-01 |
| *HES6* | Hes family bHLH transcription factor 6 | 55502 | -2.3 | 8.4E-30 | -0.7 | 1.0E-08 | -2.7 | 1.6E-42 |
| *HEY1* | Hes family bHLH transcription factor 1 | 23462 | -1.8 | 4.5E-04 | -0.7 | 1.6E-01 | -0.5 | 3.1E-01 |
| *HGF* | Hepatocyte growth factor | 3082 | -0.7 | 4.8E-01 | -1.2 | 8.6E-02 | -2.0 | 5.2E-03 |
| *HK2* | Hexokinase 2 | 3099 | -0.5 | 3.6E-04 | -0.2 | 1.8E-01 | -0.5 | 1.3E-03 |
| *IKZF1* | IKAROS family zinc finger 1 | 10320 | -0.7 | 8.4E-07 | -0.2 | 1.9E-01 | -0.6 | 8.4E-04 |
| *ITGA8* | Integrin subunit alpha 8 | 8516 | -1.0 | 5.7E-10 | -0.1 | 8.1E-01 | -0.6 | 2.6E-04 |
| *MRE11A* | MRE11 homolog A, double strand break repair nuclease | 4361 | -0.3 | 4.8E-02 | -0.2 | 2.8E-01 | -0.5 | 1.1E-02 |
| *MYB* | MYB proto-oncogene, transcription factor | 4602 | -1.7 | 3.4E-15 | -0.5 | 6.1E-04 | -2.1 | 4.5E-38 |
| *NFIL3* | Nuclear factor, interleukin 3 regulated | 4783 | -0.8 | 1.5E-11 | -0.6 | 3.8E-08 | -0.8 | 1.4E-09 |
| *NR3C2* | Nuclear receptor subfamily 3 group C member 2 | 4306 | -1.1 | 4.4E-05 | -1.1 | 1.7E-03 | -0.7 | 3.1E-02 |
| *NRAS* | Neuroblastoma RAS viral oncogene homolog | 4893 | -0.5 | 6.2E-05 | -0.2 | 3.8E-01 | -0.3 | 3.0E-02 |
| *PDE3A* | Phosphodiesterase 3A | 5139 | -0.5 | 7.7E-03 | -0.5 | 3.7E-05 | -0.9 | 5.1E-11 |
| *PDK1* | Pyruvate dehydrogenase kinase 1 | 5163 | -0.3 | 4.5E-02 | -0.1 | 5.8E-01 | -0.5 | 8.1E-04 |
| *PKP2* | Plakophilin 2 | 5318 | -0.6 | 2.9E-06 | -0.1 | 7.8E-01 | -0.5 | 2.3E-03 |
| *PPP4R4* | Protein phosphatase 4 regulatory subunit 4 | 57718 | -0.9 | 5.3E-01 | -4.4 | 5.1E-05 | -1.5 | 4.8E-02 |
| *PRDM1* | Positive regulatory domain zinc finger protein 1 | 59336 | -1.7 | 1.6E-03 | -0.1 | 9.6E-01 | -0.2 | 8.9E-01 |
| *RASGRP3* | RAS guanyl releasing protein 3 | 25780 | -0.4 | 2.3E-03 | -0.7 | 1.4E-12 | -0.1 | 6.7E-01 |
| *RBBP8* | RB binding protein 8, endonuclease | 5932 | -0.5 | 1.0E-03 | -0.1 | 6.7E-01 | -0.9 | 7.3E-09 |
| *RUNX2* | Runt related transcription factor 2 | 860 | -0.8 | 7.7E-11 | -0.3 | 8.2E-02 | -0.5 | 3.3E-02 |
| *SCG5* | Secretogranin 5 | 6447 | -0.1 | 9.1E-01 | -0.5 | 2.1E-01 | -1.1 | 2.0E-02 |
| *SIRT1* | Sirtuin 1 | 23411 | -0.4 | 9.1E-03 | -0.1 | 6.0E-01 | -0.4 | 1.7E-02 |
| *TFDP1* | Transcription factor Dp-1 | 7027 | -0.6 | 1.3E-04 | 0.0 | 9.2E-01 | -0.4 | 3.4E-03 |
| *TPST1* | Tyrosylprotein sulfotransferase 1 | 8460 | -1.0 | 3.4E-05 | -0.4 | 1.9E-01 | -0.9 | 3.6E-04 |
| *WNT5B* | Wnt family member 5B | 81029 | -0.4 | 9.6E-03 | -0.3 | 3.9E-03 | -0.5 | 1.7E-03 |

**Supplemental Table 3. List of 97-mer shRNA sequences used for the generation of the targeted shRNA library.**

For details see supplemental excel sheet.

**Supplemental Table 4. Plasmid vectors used in this study.**

| **Plasmids** | **Type of plasmid** | **Sources** |
| --- | --- | --- |
| pLKO.1-puro | Lentiviral shRNA expression vector | Addgene (Watertown, MA, USA) #8453, Bob Weinberg Lab |
| pCDH-EF1α-MCS-IRES-Neo | Lentiviral cDNA expression vector | System Biosciences (Palo Alto, CA, USA) #CD533A-2 |
| pLT3GGmirEPPIR | Lentiviral doxocycline-inducible shRNA expression vector | A derivative of the pRRL vector^4^ |
| pGIPZ | Lentiviral shRNA expression vector | Dharmacon (Lafayette, CO, USA) |
| psPAX.2 | Lentiviral packaging vector | Addgene (Watertown, MA, USA) #12260, Didier Trono lab |
| pMD2.G | Lentiviral envelope plasmid | Addgene (Watertown, MA, USA)  #12259, Didier Trono lab |
| pGL4.10[LUC2] | Promoterless firefly luciferase basic vectors | Promega (Madison, WI, USA) # E665A |
| pTK-Green Renilla Luc Vector | Green Renilla luciferase reporter vector | ThermoFisher (Watertown, MA, USA) #16154 |

**Supplemental Table 5. Antibodies used in this study.**

| **Antibodies** | **Sources** | **Catalog #** | **Applications** |
| --- | --- | --- | --- |
| Notch1 | Cell Signaling Technology | 4380 | IB, FC |
| NFIL3 | Cell Signaling Technology | 14312 | IB |
| Caspase-3 | Cell Signaling Technology | 9662 | IB |
| Cleaved Caspase-3 (Asp175) | Cell Signaling Technology | 9664 | IB |
| STAT3 | Cell Signaling Technology | 12640 | IB |
| Phospho-STAT3 (Tyr705) | Cell Signaling Technology | 9131 | IB |
| Phospho-STAT3 (Ser727) | Cell Signaling Technology | 9134 | IB |
| c-MYC | Cell Signaling Technology | 5605 | IB |
| Cyclin D1 | Cell Signaling Technology | 55506 | IB |
| CDK4 | Cell Signaling Technology | 12790 | IB |
| CDK4 | Cell Signaling Technology | 88428 | IB |
| CDK6 | Cell Signaling Technology | 3136 | IB |
| CDK6 | Cell Signaling Technology | 3048 | IB |
| CDK2 | Cell Signaling Technology | 2546 | IB |
| Histone H3 | Cell Signaling Technology | 9715 | IB |
| RBPSUH (RBPJ) | Cell Signaling Technology | 5313 | ChIP-seq |
| CD138-FITC | BioLegend | 356508 | FC |
| Rabbit IgG-Alexa Fluor 647 | Thermo Fisher Scientific | A-21245 | FC (2^nd^ Ab) |

IB, immunoblotting; FC, flow cytometry; HRP, horseradish peroxidase; 2^nd^ Ab, secondary antibody

**Supplemental Table 6. Primers for qPCR.**

| **Target genes** | **Directions** | **Sequences (5’ to 3’)** |
| --- | --- | --- |
| *NOTCH1* | Forward | ACGCACAAGGTGTCTTCCA |
| *NOTCH1* | Reverse | AGGATCAGTGGCGTCGTG |
| *HEY1* | Forward | CATACGGCAGGAGGGAAAG |
| *HEY1* | Reverse | GCATCTAGTCCTTCAATGATGCT |
| *HES1* | Forward | GGAAATGACAGTGAAGCACCT |
| *HES1* | Reverse | GGAAATGACAGTGAAGCACCT |
| *HES6* | Forward | AGCCCCTGGTGGAGAAGA |
| *HES6* | Reverse | CAGCACTTCGGCGTTCTC |
| *NFIL3* | Forward | TCCTCAGTAGAACACACGCAG |
| *NFIL3* | Reverse | TCATCTCTTGGCTCCCTTGT |
| *HPRT* | Forward | CTGGCGTCGTGATTAGTGAT |
| *HPRT* | Reverse | CTGGCGTCGTGATTAGTGAT |
| *GAPDH* | Forward | GTCTCCTCTGACTTCAACAGCG |
| *GAPDH* | Reverse | ACCACCCTGTTGCTGTAGCCAA |

HEY, Hairy/enhancer-of-split related with YRPW motif; HES, Hairy and enhancer-of-split; NFIL3, Nuclear factor, interleukin 3 regulated; HPRT, hypoxanthine–guanine phosphoribosyltransferase

**Supplemental Table 7.** Z-scores for log_2_FC in shRNA frequencies between drug- and DMSO‑treated U266-N1ΔE cells after 12 days of growth. Genes and z-scores are marked in green if values of at least two z-scores were ≤ -0.5.

|  | **U266-N1ΔE with Len** | | **U266-N1ΔE with Mel** | | **U266-N1ΔE with Btz** | |
| --- | --- | --- | --- | --- | --- | --- |
| **shRNAs against** | **Median log_2_FC** | **z-score** | **Median log_2_FC** | **z-score** | **Median log2FC** | **z-score** |
| *AFAP1L2* | -0.2 | -0.9 | 0.1 | 0.7 | 0.1 | 0.3 |
| *ATP2A3* | 0.0 | -0.2 | -0.1 | -0.4 | -0.3 | -1.2 |
| *CAD* | -0.4 | -1.5 | 0.3 | 1.2 | 0.0 | -0.3 |
| *CBFB* | -0.3 | -1.1 | -0.1 | -0.6 | 0.1 | 0.1 |
| *CCND1* | 0.1 | 0.1 | 0.2 | 1.0 | -0.1 | -0.6 |
| *CHST12* | 0.0 | -0.4 | -0.2 | -0.9 | 0.1 | 0.3 |
| *CLPTM1L* | -0.1 | -0.6 |  |  |  |  |
| *CTGF* | 0.0 | -0.3 | -0.2 | -0.9 | -0.6 | -2.4 |
| *CXCR4* | -0.1 | -0.6 | -0.1 | -0.5 | 0.2 | 0.8 |
| *DCLRE1C* | 0.0 | -0.3 | -0.3 | -1.4 | 0.0 | -0.3 |
| *DOCK10* | -0.6 | -2.1 | -0.2 | -0.7 | 0.4 | 1.6 |
| *DUSP6* | 0.2 | 0.3 | 0.2 | 1.0 | 0.1 | 0.1 |
| *ELL2* | 0.1 | -0.1 | 0.0 | 0.2 | 0.3 | 1.2 |
| *ETS1* | 0.2 | 0.5 | 0.3 | 1.3 | 0.2 | 0.7 |
| *ETV4* | 0.5 | 1.4 | 0.3 | 1.5 | -0.1 | -0.4 |
| *HES1* | -0.3 | -1.2 | 0.0 | -0.1 | -0.3 | -1.5 |
| *HES6* | 0.4 | 0.9 | 0.2 | 0.9 | 0.1 | 0.1 |
| *HEY1* | 0.6 | 1.7 | -0.2 | -0.6 | 0.0 | -0.2 |
| *HGF* | 0.1 | 0.2 | 0.1 | 0.6 | 0.3 | 0.9 |
| *HK2* | 0.1 | -0.1 | -0.2 | -0.7 | 0.3 | 0.9 |
| *IKZF1* | 0.0 | -0.2 | 0.2 | 0.7 | 0.4 | 1.6 |
| *ITGA8* | -0.1 | -0.5 | 0.1 | 0.4 | 0.0 | 0.0 |
| *Luciferase* | 0.1 | 0.0 | 0.0 | -0.1 | 0.0 | -0.4 |
| *MRE11A* | -0.5 | -1.9 | -0.1 | -0.3 | 0.0 | -0.3 |
| *MYB* | 0.4 | 0.9 | 0.1 | 0.3 | 0.1 | 0.1 |
| *NFIL3* | -0.2 | -0.7 | -0.4 | -1.7 | -0.3 | -1.4 |
| *NOTCH1* | 0.2 | 0.4 | 0.0 | 0.2 | 0.0 | -0.3 |
| *NR3C2* | 0.1 | -0.1 | 0.0 | -0.1 | -0.2 | -1.0 |
| *NRAS* | 0.2 | 0.4 | 0.3 | 1.3 | 0.3 | 1.0 |
| *PDE3A* | 0.0 | -0.3 | -0.2 | -0.8 | 0.3 | 0.8 |
| *PDK1* | 0.1 | 0.0 | -0.3 | -1.2 | -0.4 | -1.7 |
| *PKP2* | 0.1 | 0.1 | -0.2 | -0.9 | 0.0 | -0.2 |
| *PLK1* | 0.3 | 0.8 | 0.2 | 0.9 | 0.0 | -0.3 |
| *PPP4R4* | -0.4 | -1.6 | 0.3 | 1.2 | 0.4 | 1.2 |
| *PRDM1* | 0.4 | 0.9 | 0.0 | 0.2 | 0.1 | 0.1 |
| *RASGRP3* | 0.2 | 0.3 | -0.5 | -1.9 | -0.5 | -2.0 |
| *RBBP8* | 0.0 | -0.3 | -0.1 | -0.2 | 0.3 | 0.9 |
| *RUNX2* | 0.0 | -0.2 | 0.1 | 0.4 | 0.0 | -0.2 |
| *SCG5* | 0.2 | 0.4 | 0.5 | 2.3 | 0.6 | 2.2 |
| *SIRT1* | 0.4 | 1.1 | 0.1 | 0.6 | 0.2 | 0.6 |
| *TFDP1* | 1.3 | 3.7 | -0.1 | -0.3 | 0.0 | 0.0 |
| *TPST1* | 0.1 | 0.1 | -0.1 | -0.2 | 0.1 | 0.4 |
| *UBB* | 0.3 | 0.6 | 0.0 | -0.1 | 0.1 | 0.2 |
| *WNT5B* | 0.2 | 0.4 | -0.6 | -2.6 | -0.3 | -1.4 |

**Supplemental Table 8. MM cell lines and patient characteristics with high-risk cytogenetics.**

For details see supplemental excel sheet.

References: ^12-16^

**Supplemental Figures**

**Supplemental Figure 1**

shNTC

DMSO

0

0.5

1

1.5

Fold change

shN1 #1

shN1 #2

*HEY1*

*HES6*

GSI XII

MM.1S

MM.1S

***

***

***

***

***

**

***

***

**

**

Fold change

*HES6*

*HES1*

*HEY1*

*NOTCH1*

**Supplemental Figure 1.** **Downregulation of Notch target genes in MM.1S cells after Notch inhibition through GSI XII or *NOTCH1* knockdown.** Quantitative PCR analysis of the expression of *NOTCH1* and the target genes *HEY1*, *HES1*, and *HES6* in MM.1S cells after Notch inhibition and *NOTCH1* knockdown. Data are shown as mean ± SD of a representative experiment, each dot representing a technical replicate (n ≥ 3). For statistical analysis, unpaired *t*-tests were used. ***P*-value ≤ 0.01, ****P*-value ≤ 0.001.

**Supplemental Figure 2**

0

0.5

1

1.5

*HES6*

*HES1*

*HEY1*

*NOTCH1*

Fold change

**Supplemental Figure 2. Targeted functional genomic screen in U266 cells**. Histogram showing the representation of the shRNA sequences in the pooled library on plasmid level as quality control.

**Supplemental Figure 3**

**Supplemental Figure 3. Overexpression of the Notch1 variant N1ΔE in U266 cells.** Histograms for Notch1 expression in U266-vector and U266-N1ΔE cells detected by intracellular flow cytometry analysis. A647: Alexa Fluor 647.

**Supplemental Figure 4**

Notch1

-

ΔE

ΔE

**A**

**Supplemental Figure 4. Effects of knockdown and depletion of NFIL3 in U266, MM.1S and MOPC315.BM cells.** (A) Immunoblots with whole extracts of U266 cells expressing the empty vector (-) or the Notch1 variant N1ΔE (ΔE) with or without knockdown of NFIL3 (NFIL3 sh#1). (B) Immunoblots with whole extracts of MM.1S cells after knockdown with shNFIL3 #1 or shNFIL3 #2. Blots were probed with antibodies against pStat3 (Y705), pStat3 (S727), Stat3, and Histone H3. (C) Immunoblots with whole extracts of MM.1S cells after treatment with RA for 24 h. Blots were probed with antibodies against pStat3 (Y705), pStat3 (S727), Stat3, and Histone H3. (D) Immunoblots with whole extracts of MOPC.315.BM.Luc.eGFP-mCrbnI^391V^ cells after treatment with RA for 24 h. Blots were probed with antibodies against pStat3 (Y705), pStat3 (S727), Stat3, and Histone H3. Quantification was performed for three to five biological replicates using ImageJ. *T*-tests were used for statistical analysis. **P*-value ≤ 0.05, ***P*-value ≤ 0.01, ****P*-value ≤ 0.001.

shNFIL3 #1

0

50

100

150

pStat3 (S727)/H3

*

*

Relative expression

Relative expression

Relative expression

0

50

100

150

Stat3/H3

**

***

0

50

100

150

pStat3(Y705)/H3

**

**

Relative expression

Relative expression

Relative expression

0

50

100

150

pStat3 (S727)/H3

**ns**

**ns**

*

***

0

50

100

150

200

pStat3 (Y705)/H3

**ns**

**ns**

**ns**

**ns**

0

50

100

150

200

Stat3/H3

*

**ns**

**ns**

**ns**

shNTC

shNFIL3 #1

shNFIL3 #2

0

50

100

150

pStat3 (Y705)/H3

**

**

***

0

50

100

150

pStat3 (S727)/H3

**

****

****

Relative expression

Relative expression

Relative expression

0

50

100

150

Stat3/H3

**ns**

**

**ns**

RA (µM)

0

1

1.5

2

pStat3 (Y705)

Stat3

pStat3 (S727)

Histone H3

MOPC

DMSO

1 µM RA

2 µM RA

3 µM RA

4 µM RA

Histone H3

MM.1S

pStat3 (S727)

Stat3

pStat3 (Y705)

Histone H3

shNFIL3

-

1

2

c-Myc

-

-

+

NFIL3

Notch1

pStat3 (Y705)

Stat3

pStat3 (S727)

Histone H3

RA [µM]

0	1	2	3	4

MM.1S

**C**

DMSO

1 µM RA

1 µM RA

1.5 µM RA

**D**

**B**

**Supplemental Figure 5**

Cell viability [%]

0

50

100

150

LP-1

3 µM Mel

DMSO

10 µM Len

4 nM Btz

Cell viability [%]

0

50

100

150

RPMI 8226

1.75 µM Mel

DMSO

10 µM Len

1.5 nM Btz

shNTC

shNFIL3 #1

shNFIL3 #2

shNTC

shNFIL3 #1

shNFIL3 #2

**Supplemental Figure 5. Knockdown of NFIL3 reduces viability of RPMI and LP-1 cells.** Cell viability of human MM cells (RPMI 8226, LP-1) after knockdown of *NFIL3* (shNFIL3 #1, shNFIL3 #2) and treatment with Len, Mel, and Btz for three days at the indicated concentrations was determined using the CellTiter-Glo® Luminescent Cell Viability Assay. Data are presented as the mean ± SD of a representative experiment, each dot representing a technical replicate (n ≥ 3). An unpaired *t*-test was used for statistical analysis. *P-value ≤ 0.05, **P-value ≤ 0.01, ***P‑value ≤ 0.001.

**Supplemental Figure 6**

**A**

MOPC.315.BM.Luc.eGFP-*mCrbn^I391V^*

**B**

**D**

**C**

2

20

40

0

4

6

DMSO

3 µM RA

4 µM Mel

3 µM RA +
4 µM Mel

DMSO

2 µM RA

4 µM Mel

2 µM RA +
4 µM Mel

RPMI 8226

RPMI 8226

Cell viability [%]

0

50

100

150

DMSO

2 µM RA

10 µM Len

2 µM RA +
10 µM Len

Cell viability [%]

0

50

100

150

Cell viability [%]

0

50

100

150

DMSO

2 µM RA

10 nM Btz

2 µM RA +
10 nM Btz

Cell viability [%]

0

50

100

150

DMSO

3 µM RA

10 µM Len

3 µM RA +
10 µM Len

Cell viability [%]

0

50

100

150

Cell viability [%]

0

50

100

150

DMSO

3 µM RA

3 nM Btz

3 µM RA +
3 nM Btz

***

**

Cell number [x 10^7^]

***

**

**

***

0

3

6

9

12

15

Time [days]

***

***

***

***

**

0.75 µM RA + 2 µM Len

DMSO

0.75 µM RA

2 µM Len

2

20

40

0

4

6

**

**

Cell number [x 10^7^]

**

***

***

0

3

6

9

12

15

Time [days]

***

***

**

**

***

0.75 µM RA + 1 µM Mel

DMSO

0.75 µM RA

1 µM Mel

2

20

40

0

4

6

Cell number [x 10^7^]

0

3

6

9

12

15

Time [days]

0.75 µM RA + 2 nM Btz

DMSO

0.75 µM RA

2 nM Btz

*

***

***

**

***

***

*

**

***

**

**

**

**

**

***

***

**

***

***

**Supplemental Figure 6. RA sensitizes MM cell lines to Len, Mel, and Btz.** (A) Cell viability of the human MM cell lines OPM-2, LP-1, IM-9, AMO-1, RPMI 8226, MM.1S, the engineered cell lines U266-vector and U266-N1ΔE, and the murine cell line MOPC.315.BM.Luc.eGFP-*mCrbn^I391V^* upon treatment with RA for 48 h. Data are presented as mean ± SD of at least three independent experiments. (B) Cell viability of MOPC.315.BM.Luc.eGFP-*mCrbn^I391V^* cells after treatment with DMSO, RA, Len, Mel, and Btz in concentrations as indicated for 48 h. Data are given as the mean ± SD of three independent experiments (shown with dots). An unpaired *t*-test was used for statistical analysis. (C) Cell viability of RPMI 8226 cells after treatment with RA, Len, Mel, and Btz as single treatments or in combination with the indicated concentrations for six days. Data are represented as mean ± SD of three independent experiments; each dot represents a replicate. For statistical analysis, unpaired *t*-tests were used. (D) Cell counts of RPMI 8226 cells grown in media containing the indicated concentrations of the drugs for 15 days. The number of viable cells was determined every three days using trypan blue. Subsequently, cells were reseeded to the initial cell number of 3 × 10^5^ cells/ml. The three line graphs share the same DMSO and RA datasets. Data are presented as mean ± SD of one representative experiment from at least three independent experiments. For statistical analysis, unpaired *t*-test was used to compare between drug-treated cells (RA alone or in combination with Len, Mel, or Btz) and the control cells (DMSO). **P*‑value ≤ 0.05, ***P*-value ≤ 0.01, ****P*-value ≤ 0.001.

**Supplemental Figure 7**

**A**

**D**

**C**

**B**

**Supplemental Figure 7. Drug quantification.** (A) Chemical structures of the drugs RA (PubChem CID 174742)^17^ and Len (PubChem CID 216326)^17^ used for formulation development. (B) Representative HPLC chromatograms show the typical peaks and retention times for RA at 6.05 min and Len at 7.52 min. (C) HPLC solvent gradients for RA and Len in [%] ACN/H2O. (D) Increased dilutions of the free drugs in methanol were measured in triplicates by HPLC to obtain the standard curves. Standard curves show the mean ± SD of the determined area peaks, and obtained equations were used to calculate the RA and Len concentrations. For both drugs, drug formulations were developed using the thin film hydration method. The increase in solubilized drug, observed with the increasing drug feed (supplemental Figure 7), was quantified with HPLC, as shown here.

**Supplemental Figure 8**

**Supplemental Figure 8. Polymer cytocompatibility test in vitro.** Cell viability of MM cells (MM.1S, RPMI 8226, AMO-1, MOPC315.BM.Luc.eGFP-*mCrbn^I391V^*) upon incubation in medium containing the indicated concentrations of the plain polymer A-pBuOzi-A for 48 h. Data are presented as mean ± SD of six technical replicates. A-pBuOzi-A was found to be highly cytocompatible (cell viability 100 %) in MM.1S, RPMI 8226, AMO-1, and MOPC315.BM.Luc.eGFP-*mCrbn^I391V^* cells up to a concentration of 1.25 g/l, while a slight decrease in viability (80 % of viability) was observed at concentrations of 2.5 and 5 g/l in MM.1S, RPMI 8226, and MOPC315.BM.Luc.eGFP-*mCrbn^I391V^* cells. Notably, these concentrations are approx. 100 times higher than polymer concentrations used in drug formulations (supplemental Figure 9).

**Supplemental Figure 9**

**A**

**C**

**B**

0 µM RA, 0 µM Len

2 µM RA

0.16 µM Len

2 µM RA + 0.16 µM Len

Cell viability [%]

0

50

100

150

DMSO

Polymer

Cell viability [%]

0

50

100

150

Cell viability [%]

0

50

100

150

DMSO (0.010 %)

Polymer (0.013 g/l)

DMSO (0.012 %)

Polymer (0.013 g/l)

0 µM

0.16 µM

1.25 µM

10 µM

[Len]

0 µM

0.15 µM

2 µM

3 µM

[RA]

**D**

**E**

**Supplemental Figure 9. Micellar drug nanoformulations using the ABA triblock copolymer A-pBuOzi-A and in vitro cytotoxic effects of drug-loaded polymer micelles.** (A) Solubilized aqueous concentration of RA and Len formulated A-pBuOzi-A in dependence of the drug feed. Polymer feed was 10 g/l, and drug feed was varied from 1 to 10 g/l. For an initial stability assessment, the aqueous formulations were stored at ambient conditions containing the initial precipitate (if any) after thin film hydration. The samples were collected at day 0, 1, 5, and 15 with centrifugation before each collection to sediment precipitate, if any. The soluble drug remaining in the supernatant was then quantified by HPLC. (B) Achieved loading capacity (LC, equation 1) and (C) loading efficiency (LE, equation 2) of A-pBuOzi-A at constant polymer feed of 10 g/l, with increasing drug feed from 1 to 10 g/l, measured on the indicated days is reported. Data is given as means ± SD (n = 3). For both drugs, an increase in LC was observed with increasing drug feed. The triblock copolymer A-pBuOzi-A was capable to solubilize 10 g/l RA (LC = 50 weight %) and 3 g/l Len (LC = 23 weight %), respectively. Once prepared, all formulations were stored as lyophilized powder, followed by redispersion with PBS when needed. (D) Cell viability of MOPC.315.BM.Luc.eGFP-*mCrbn^I391V^* cells treated with RA- and Len-loaded micelles vs empty micelles compared to the free drugs (Len, RA vs DMSO) at the indicated concentrations for 48 h. (E) Cell viability after single and combination treatment as indicated. Data is presented as means ± SD of three independent experiments. Unpaired *t*-tests were used for statistical analysis. ***P*-value ≤ 0.01, ****P*-value ≤ 0.001.

**Supplemental Figure 10**

0

8

6

4

2

Control

Vehicle

RA

Len

RA/Len

0

20

BV/TV [%]

60

40

Control

Vehicle

RA

Len

RA/Len

0

10

OcS/BS [%]

30

20

Control

Vehicle

RA

Len

RA/Len

Lesion score

**A**

**B**

**C**

**Supplemental Figure 10. Effects of RA and Len on osteolytic bone disease in MM mice.** Seven days after i.t. injection of 2.5 × 10^4^ MOPC.315.BM.Luc.eGFP-mCrbnI391V cells or PBS (control), mice were treated with vehicle (polymer), 3 mg/kg RA, 5 mg/kg Len, or the combination five times per week for three weeks. On day 26, mice were sacrificed, and the tibiae were extracted and fixed. Using microCT analysis and tartrate-resistant acid phosphatase (TRAP) staining, lesion scores (A), trabecular bone volume to total volume fraction (BV/TV; B), and osteoclast surface to bone surface (OcS/BS; C) of tibiae were determined. Values are shown as the mean ± SEM. An ANOVA test with Tukey’s post hoc correlation was used for statistical analysis. **P*-value ≤ 0.05, ***P*-value ≤ 0.01,667, *****P*-value ≤ 0.0001.

**Supplemental Figure 11**

DMSO

RA

Mel

RA + Mel

DMSO

RA

Btz

RA + Btz

DMSO

RA

Len

RA + Len

Cell viability [%]

0

50

100

150

200

Cell viability [%]

0

50

100

150

200

Cell viability [%]

0

50

100

150

200

Pt. MM #5

Pt. MM #8

Pt. MM #10

Pt. MM #6

Pt. MM #11

Pt. MM #13

Pt. MM #14

Pt. MM #15

Pt. MM #5

Pt. MM #8

Pt. MM #10

Pt. MM #6

Pt. MM #11

Pt. MM #13

Pt. MM #14

Pt. MM #15

Pt. MM #5

Pt. MM #8

Pt. MM #10

Pt. MM #6

Pt. MM #11

Pt. MM #13

Pt. MM #14

Pt. MM #15

***

***

***

*

*

*

*

**

**

***

***

***

***

***

*

***

***

***

***

***

***

**

**

**

***

***

***

**

**

*

***

***

***

*

*

**

***

***

***

***

***

**

***

***

***

*

***

***

***

**

***

***

***

**

**

*

**

**

*

*

**

**

***

***

***

***

***

***

*

***

***

***

***

***

**

***

***

***

***

***

***

***

**

**

**

**

**

*

***

***

***

***

**Supplemental Figure 11. RA kills multi-refractory high-risk patient MM cells and shows additive effects with Len, Mel, and Btz.** Cell viability of primary MM cells treated with DSMO, RA (2 µM), Len (2 µM), Mel (2 µM), and Btz (3 nM) for 48 h. Viability was determined using the CellTiter-Glo® Luminescent Cell Viability Assay. Unpaired *t*-tests were used for statistical analysis. Data are presented as the mean ± SD of technical triplicates. **P*‑value ≤ 0.05, ***P*-value ≤ 0.01, ****P*-value ≤ 0.001.

**Supplemental Figure 12**

Pt. CD34^+^ #16

Pt. CD34^+^ #17

Pt. CD34^+^ #18

Cell viability [%]

0

50

100

150

DMSO

2 µM RA

***

*

**Supplemental Figure 12.**

Treatment of CD34-positive primary stem cells with DMSO or 2 µM RA for 48 h shows reduced toxicity of RA for stem cells. Viability was determined using the CellTiter-Glo® Luminescent Cell Viability Assay. Unpaired *t*-tests were used for statistical analysis. Data are presented as the mean ± SD of technical triplicates. **P*‑value ≤ 0.05, ****P*-value ≤ 0.001.

**References**

1.	Ilagan MX, Lim S, Fulbright M, Piwnica-Worms D, Kopan R. Real-time imaging of notch activation with a luciferase complementation-based reporter. *Sci Signal*. 2011;4(181):rs7. DOI: 10.1126/scisignal.2001656.

2.	Oliveros JC. An Interactive Tool for Comparing Lists with Venn’s Diagrams. <https://bioinfogp.cnb.csic.es/tools/venny/index.html>. 2007-2015.

3.	Papadopoulos D, Ade CP, Eilers M. Generation of a pooled shRNA library for functional genomics screens. *STAR Protoc*. 2022;3(1):101183. DOI: 10.1016/j.xpro.2022.101183.

4.	Fellmann C, Hoffmann T, Sridhar V, et al. An optimized microRNA backbone for effective single-copy RNAi. *Cell Rep*. 2013;5(6):1704-1713. DOI: 10.1016/j.celrep.2013.11.020.

5.	Pelossof R, Fairchild L, Huang CH, et al. Prediction of potent shRNAs with a sequential classification algorithm. *Nat Biotechnol*. 2017;35(4):350-353. DOI: 10.1038/nbt.3807.

6.	Jung LA, Gebhardt A, Koelmel W, et al. OmoMYC blunts promoter invasion by oncogenic MYC to inhibit gene expression characteristic of MYC-dependent tumors. *Oncogene*. 2017;36(14):1911-1924. DOI: 10.1038/onc.2016.354.

7.	Langmead B. Aligning short sequencing reads with Bowtie. *Curr Protoc Bioinformatics*. 2010;Chapter 11:Unit 11 17. DOI: 10.1002/0471250953.bi1107s32.

8.	Rohner L, Ng YLD, Scheffold A, et al. Generation of a lenalidomide-sensitive syngeneic murine in vivo multiple myeloma model by expression of Crbn(I391V). *Exp Hematol*. 2021;93:61-69 e64. DOI: 10.1016/j.exphem.2020.11.004.

9.	Lubtow MM, Oerter S, Quader S, et al. In Vitro Blood-Brain Barrier Permeability and Cytotoxicity of an Atorvastatin-Loaded Nanoformulation Against Glioblastoma in 2D and 3D Models. *Mol Pharm*. 2020;17(6):1835-1847. DOI: 10.1021/acs.molpharmaceut.9b01117.

10.	Lubtow MM, Nelke LC, Seifert J, et al. Drug induced micellization into ultra-high capacity and stable curcumin nanoformulations: Physico-chemical characterization and evaluation in 2D and 3D in vitro models. *J Control Release*. 2019;303:162-180. DOI: 10.1016/j.jconrel.2019.04.014.

11.	Bouxsein ML, Boyd SK, Christiansen BA, Guldberg RE, Jepsen KJ, Muller R. Guidelines for assessment of bone microstructure in rodents using micro-computed tomography. *J Bone Miner Res*. 2010;25(7):1468-1486. DOI: 10.1002/jbmr.141.

12.	Chesi M, Bergsagel PL, Shonukan OO, et al. Frequent dysregulation of the c-maf proto-oncogene at 16q23 by translocation to an Ig locus in multiple myeloma. *Blood*. 1998;91(12):4457-4463.

13.	Katagiri S, Yonezawa T, Kuyama J, et al. Two distinct human myeloma cell lines originating from one patient with myeloma. *Int J Cancer*. 1985;36(2):241-246. DOI: 10.1002/ijc.2910360217.

14.	Shepard JS, Pettengill OS, Wurster-Hill DH, Sorenson GD. Karyotype, marker formation, and oncogenicity in mouse plasmacytomas. *J Natl Cancer Inst*. 1976;56(5):1003-1011. DOI: 10.1093/jnci/56.5.1003.

15.	Bergsagel PL, Chesi M, Nardini E, Brents LA, Kirby SL, Kuehl WM. Promiscuous translocations into immunoglobulin heavy chain switch regions in multiple myeloma. *Proc Natl Acad Sci U S A*. 1996;93(24):13931-13936. DOI: 10.1073/pnas.93.24.13931.

16.	Drexler HG, Matsuo Y. Malignant hematopoietic cell lines: in vitro models for the study of multiple myeloma and plasma cell leukemia. *Leuk Res*. 2000;24(8):681-703. DOI: 10.1016/s0145-2126(99)00195-2.

17.	Kim S, Chen J, Cheng T, et al. PubChem in 2021: new data content and improved web interfaces. *Nucleic Acids Res*. 2021;49(D1):D1388-D1395. DOI: 10.1093/nar/gkaa971.
